# Does integument arise *de novo* or from pre-existing structures? ── Insights from the key regulatory genes controlling integument development

**DOI:** 10.3389/fpls.2022.1078248

**Published:** 2023-01-13

**Authors:** Min Jiang, Jinjing Jian, Chengchuan Zhou, Linfeng Li, Yuguo Wang, Wenju Zhang, Zhiping Song, Ji Yang

**Affiliations:** ^1^ Ministry of Education Key Laboratory for Biodiversity Science and Ecological Engineering, Center for Evolutionary Biology, Fudan University, Shanghai, China; ^2^ Shanghai Key Laboratory of Plant Functional Genomics and Resources, Shanghai Chenshan Botanical Garden, Shanghai, China; ^3^ Institute of Tree Genetics Breeding and Cultivation, Jiangxi Academy of Forestry, Nanchang, China

**Keywords:** seed formation, integument origin, successive transformation, regulatory gene, molecular evolution, evo-devo, serial homology

## Abstract

The origin of seeds is one of the key innovations in land plant evolution. Ovules are the developmental precursors of seeds. The integument is the envelope structure surrounding the nucellus within the ovule and developing into the seed coat when ovules mature upon fertilization. The question of whether the integument arise *de novo* or evolve from elaboration of pre-existing structures has caused much debate. By exploring the origin and evolution of the key regulatory genes controlling integument development and their functions during both individual and historical developmental processes, we showed the widespread presence of the homologs of *ANT*, *CUC*, *BEL1*, *SPL*, *C3HDZ*, *INO*, *ATS*, and *ETT* in seedless plant genomes. All of these genes have undergone duplication-divergence events in their evolutionary history, with most of the descendant paralogous suffering motif gain and/or loss in the coding regions. Expression and functional characterization have shown that these genes are key components of the genetic program that patterns leaf-like lateral organs. Serial homology can thus be postulated between integuments and other lateral organs in terms of the shared master regulatory genes. Given that the genetic program patterning leaf-like lateral organs formed in seedless plants, and was reused during seed origin, the integument is unlikely to arise *de novo* but evolved from the stem segment-specific modification of pre-existing serially homologous structures. The master ‘switches’ trigging the modification to specify the integument identity remain unclear. We propose a successive transformation model of integument origin.

## Background

Seed formation is one of the key innovations in land plant evolution (Linkies et al., 2010; Baroux and Grossniklaus, 2019; Meade et al., 2021). The protective and nourishing structures of the seed enable a maternal care of the young sporophyte (embryo) and ensure seed dispersal over large areas for long time spans to colonize different environments, fueling seed plant radiation (Baroux and Grossniklaus, 2019). The origin of the seed is not a single innovation, but a unique ovule-centered reproductive syndrome involving a set of innovations that define the seed habit (Gerrienne and Meyer-Berthaud, 2007; Meyer-Berthaud et al., 2018; Hetherington, 2021; Meyer-Berthaud, 2022). The ovule is the developmental precursor of the seed. The molecular regulation mechanisms of ovule development have been systematically studied in model plants, such as *Arabidopsis thaliana* (Gasser et al., 1998; Gasser and Skinner, 2019; Barro-Trastoy et al., 2020). However, the comprehensive analysis of the evolutionary origin of the specialized structures of ovule that contain and protect the developing embryos is still limited, including the origin and evolution of the maternally derived integument — the one- or two-layer envelope surrounding the nucellus and developing into the seed coat when ovules mature after fertilization (D'Apice et al., 2021).

The integument is an important structure within the ovule, functioning to protect the internal tissues and define a route *via* the micropyle for the transfer of sperm from the pollen tube to the ovum (Linkies et al., 2010; Lora et al., 2019). The question of how the integument originate has caused much debate. Diverse theories have been put forward to explain the origin of integument. The telome theory is the most acceptable, which portrays the evolution of the integument through fusion of sterile branches or telomes around a terminal megasporangium (Herr, 1995). Distinct from the transformational hypotheses that integuments have originated through modification of pre-existing ancestral structures, such as the dichotomously branching axes (Zimmermann, 1952; Andrews, 1963; Smith, 1964) or sterilized sporangia of a synangium (Kenrick and Crane, 1997), an alternative hypothesis has been proposed based on developmental and genetic evidence, which suggests that ovules have characteristics of meristems (Gross-Hardt et al., 2002), and that integuments are lateral organs initiated by the nucellar meristems and are of *de novo* origin (Mathews and Kramer, 2012; Hetherington, 2021).

**Figure 1:**
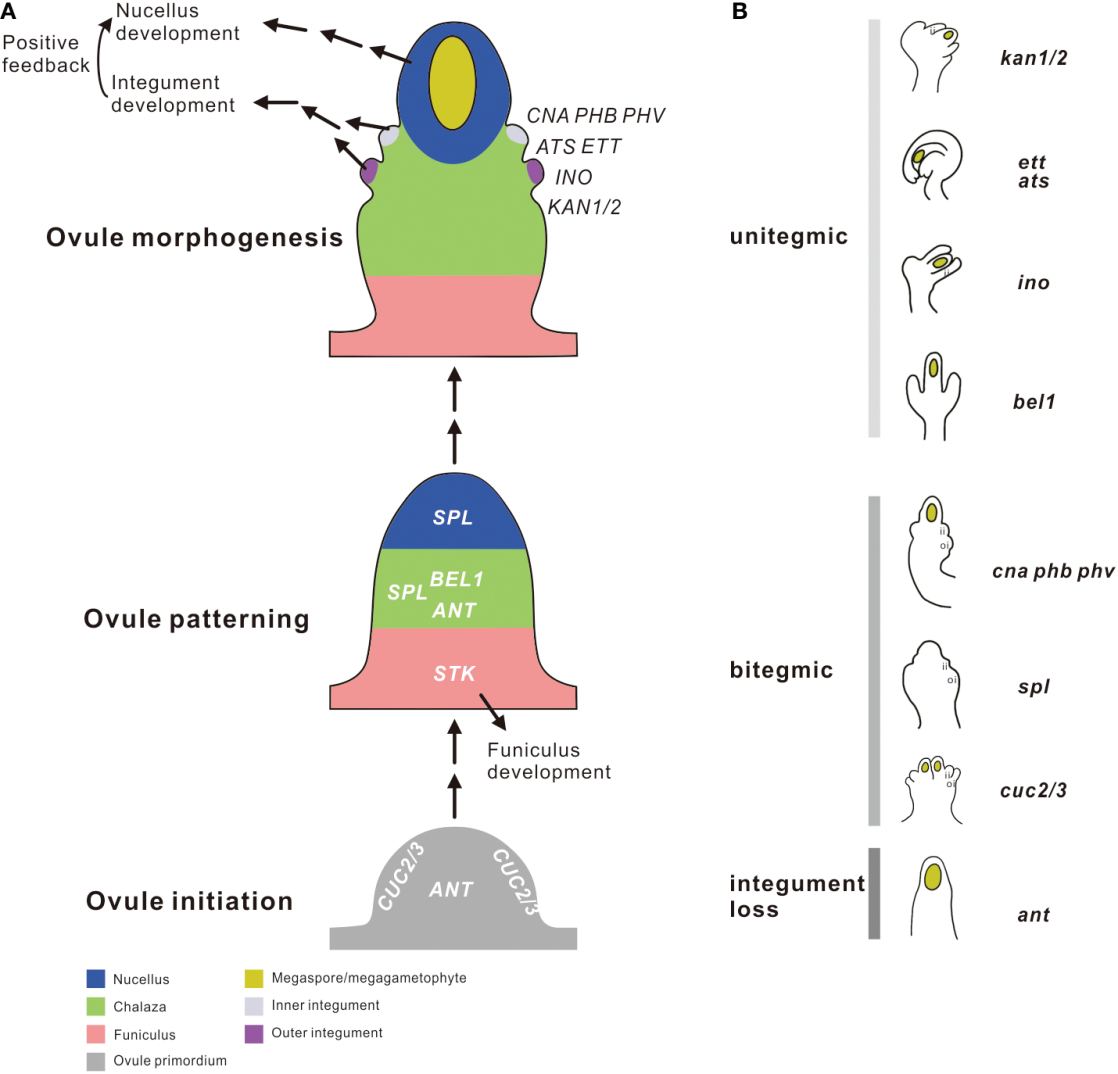
Schematic diagram demonstrating the genes regulating integument growth at different stages of ovule development. **(A)** The process of ovule development is divided into three stages in a bottom-up order: ovule initiation, ovule patterning, and ovule morphogenesis. Different tissue zones of the ovule are illustrated with different colors. Gene names are shown in the sites and developmental stages they are believed to act. **(B)** The integument phenotypes of different mutants. Refer to text for detailed explanation for each mutant. ii, inner integument; oi, outer integument; i, integument; unitegmic, possessing only a single integument; bitegmic, possessing two distinct integuments; integument loss, complete loss of two distinct integuments.

The paleontological perspective of the nature and origin of the integument is apparently inconsistent with the hypothesis deduced from the developmental genetic analysis of extant plants. The accumulated knowledge on genes and pathways involved in integument development, as well as the advent of massive genomic data from a wide range of species, opens the possibility to use master regulatory genes as markers to identify historically *vs* serially homologous structures by detecting the presence of well-characterized integument developmental genes throughout the evolutionary timeline and exploring whether the structures with different morphologies have been orchestrated by the same developmental system (Jaramillo and Kramer, 2007; Tomoyasu et al., 2017). Given the prominent role of transcription factors in driving morphological innovations, exploring the evolution of key transcription factor genes constructing the integument gene network may contribute to bridging the disconnects between fossils and genes (Zumajo-Cardona and Ambrose, 2020; Tomescu and Rothwell, 2022). Functional studies in the model species *A. thaliana* have identified a dozen transcription factors genes directing integument formation at different stages of ovule development, including ovule primordium initiation (*ANT* and *CUC*), ovule patterning (*BEL1*, *SPL*/*NZZ* and *STK*) and ovule morphogenesis (*C3HDZ*, *INO*, *KAN*, and *ETT*) (**Figure 1**). Of them, *AINTEGUMENTA* (*ANT*), a member of the *APETALA2* (*AP2*) transcription factor gene family, participates in the positive regulation of the ovule and integument primordia initiation (Elliott et al., 1996). Single *ant* mutation can lead to the complete loss of integuments, as well as the reduction in ovule number with no concomitant reduction in pistil length (Elliott et al., 1996; Klucher et al., 1996). The two *CUP SHAPED COTYLEDON* genes (*CUC2* and *CUC3*) that encode the transcription factors of the NAC family coordinate the pattern formation of ovules, and are mainly involved in the establishment of ovule primordia boundaries. The *cuc2* and *cuc3* double mutant harbors defects in ovule separation, producing fused ovule primordia with shared integuments that ultimately form fused seeds sharing seed coat (Galbiati et al., 2013; Goncalves et al., 2015). Mutations in *CUC1* and *CUC2* also lead to abnormal ovule spacing, which affects the number of normal ovule (Goncalves et al., 2015). The *BELL1* (*BEL1*) gene encodes a homeodomain protein involved in the initiation of integument development. The *bel1* mutant shows significant growth in the chalazal region where an amorphous structure develops instead of integuments (Robinsonbeers et al., 1992; Brambilla et al., 2007). *SPOROCYTELESS*/*NOZZLE* (*SPL*/*NZZ*) belongs to a transcription repressor family that is specific in embryophyte, promoting the formation of megasporocyte and integuments during ovule development (Wei et al., 2015). It has been shown that *SPL*/*NZZ* interacts with *BEL1* to control chalaza and integument development. In the *bel1* and *spl*/*nzz* double mutant, the ovules developed as finger-like structures without integuments (Balasubramanian and Schneitz, 2002; Bencivenga et al., 2012). It is noteworthy that SEEDSTICK (STK) is a negative regulator of funiculus development, and its mutants show drastically enlargement of the funiculi (Pinyopich et al., 2003). The adaxial functions of the class III homeodomain leucine zipper (*C3HDZ*) genes and the abaxial functions of the *YABBY* (*YAB*) and *KANADI* (*KAN*) family genes contribute to the integument shape and outgrowth. However, there exist differences in how these genes participate in this interaction (Gasser and Skinner, 2019). The *C3HDZ* genes *PHABULOSA* (*PHB*)*, PHAVOLUTA* (*PHV*) and *CORONA* (*CNA*) are expressed in the adaxial layer of the inner integument, playing roles in defining adaxial regions of the planar integuments and causing reduced growth in both integuments when multiple family members are mutated (Kelley et al., 2009; Yamada et al., 2016). The other *C3HDZ* gene *REVOLUTA* (*REV*) is expressed across both integuments, promoting adaxial activity in the outer as well as inner integument (Kelley et al., 2009). *INNER NO OUTER* (*INO*) is the only *YAB* gene expressed in ovules and is essential for the formation of outer integuments (Villanueva et al., 1999). *INO* is expressed in the abaxial domains of the outer integument and its mutation leads to a complete absence of the outer integument (Villanueva et al., 1999). Recently it was shown that *STIMPY* (*STIP*; also known as *WUSCHEL-RELATED HOMEOBOX 9*) can regulate *INO*, functioning as a regulator of outer integument formation (Petrella et al., 2022). The *KAN* family genes *KAN1* and *KAN2* are also expressed in the abaxial region of the outer integument (McAbee et al., 2006). The *kan1* and *kan2* double mutant grows an amorphous structure in place of the outer integument (Eshed et al., 2001; McAbee et al., 2006). Another *KAN* gene, *ABERRANT TESTA SHAPE* (*ATS*) (also called *KAN4*), functions in regulating the inner integument development and the separation of two integuments. The *ats* mutants have a single integument, which is formed by fusing the inner and outer integuments (McAbee et al., 2006). *ETTIN* (*ETT*) (also called *AUXIN RESPONSE FACTOR 3*, *ARF3*) encodes a transcription factor physically interacting with ATS to define the boundary between integument primordia. Mutation of *ETT* generates the same integument fusion phenotype as seen in the *ats* mutant (Kelley et al., 2012).

The transcription factor genes mentioned above together establish a regulatory blueprint for integument primordia initiation and outgrowth during ovule development (Schneitz et al., 1995) (**Figure 1**). Although their functions have been extensively evaluated in different angiosperms with divergent ovule numbers and morphology (Yamada et al., 2001; Brown et al., 2010; Lora et al., 2011; Lora et al., 2015), questions remain unclear about how these genes evolved, how the timing of their appearance relates to the origination of integuments, and whether the wiring of these genes have changed over evolutionary time? In this study, we reconstructed the evolutionary history of these genes based on accumulated whole genome sequences. By examining the taxonomic distribution, structural diversification following duplication, and the spatiotemporal expression of these genes reported in diverse seed and seedless plants, we aim to extend our understanding of the function of the core regulatory genes described in model plants to ancestral lineages prior to the angiosperm diversification, to approach the fundamental nature of integuments and evaluate the competing explanations for the origin of the integument.

## Materials and methods

### Genome-wide identification and phylogenetic analysis

*ANT* belongs to the *AP2*/*ERF* gene family that are divided into two classes based on the number of the AP2 domain (repeated units 1 and 2, abbreviated as R1 and R2): the *AP2*-like class containing two AP2 domains and the *ERF*-like class containing one AP2 domain (Kim et al., 2006). *AP2*-like genes are further divided into two groups: the *ANT* group has a 10-aa insertion in the R1 domain and the *euAP2* group without insertion. The *ANT* group consists of two subgroups: *euANT* and *basalANT*. There exist 10 *ANT* group members in the *A. thaliana* genome, which were used as queries to perform BLASTP searches against 49 plant genomes and transcriptomes (**Supplementary Table S1**) with an E-value threshold of 1e^-5^. The hit sequences with similarity to the query sequence were annotated with InterProScan (https://www.msi.umn.edu/sw/interproscan) (Quevillon et al., 2005), and sequences containing two conserved AP2 domains (R1 and R2) were retained and used for the next analysis. All the sequences were aligned using MAFFT v7.471 (Katoh and Standley, 2013) and trimmed by trimAL v1.4.1 (Capella-Gutierrez et al., 2009) with the threshold of -gt = 0.9 and -cons = 30. The phylogenetic tree was reconstructed using IQ-TREE (Nguyen et al., 2015) based on the JTT+G+F model selected by ProtTest3 (Darriba et al., 2011) with 2000 bootstraps.

The *CUC1*, *CUC2* and *CUC3* genes encode the NAC domain proteins, belonging to the subgroup II-3 of the NAC superfamily (Vroemen et al., 2003; Pereira-Santana et al., 2015; Maugarny-Cales et al., 2016). There are 11 members in the *A. thaliana* genome (Jensen et al., 2010), which were used as probes to perform BLASTP searches against 49 plant genomes and transcriptomes with an E-value threshold of 1e^-50^ (**Supplementary Table S1**). Sequences annotated to contain “NAC_dom” with InterProScan being retained and used for the next analysis. The NAC domain contain five conserved motifs: A, B, C, D and E (Puranik et al., 2012). All sequences were aligned using MAFFT v7.471 and trimmed using trimAL v1.4.1 with the thresholds of -gt = 0.9 and -cons = 25. The Maximum Likelihood (ML) phylogenetic tree was reconstructed using IQ-TREE with the JTT+I+G model selected by ProtTest3 with 2000 bootstraps.

*BEL1* is a member of the *BEL1*-like homeodomain (*BLH*) family of the *TALE* (Three Amino acid Loop Extension) homeodomain superfamily (Mukherjee et al., 2009). There exist 13 *BLH* members in the *A. thaliana* genome, which were used as queries to perform BLASTP searches against 49 plant genomes and transcriptomes (**Supplementary Table S1**) with an E-value threshold of 1e^-5^. Sequences containing both ‘‘POX_dom’’ and “Homeobox_dom” were retained and used for the next analysis. The POX domain is composed of SKY and BEL-B domains (Mukherjee et al., 2009). All sequences were aligned using MAFFT v7.471 and trimmed by trimAL v1.4.1 with the threshold of -gt = 0.5. The ML phylogenetic tree was reconstructed using IQ-TREE based on the JTT+G+F model selected by ProtTest3 with 2000 bootstraps.

*SPL*/*NZZ* belongs to the *SPEAR* (SPL-like, EAR-containing proteins) family (Chen et al., 2014). There are five *SPEAR* members in *A. thaliana* genome, which were used as queries to perform BLASTP searches with an E-value threshold of 0.5 against 49 plant genomes and transcriptomes (**Supplementary Table S1**). The sequences were further annotated with InterProScan with the sequences containing the ‘‘NOZZLE’’ domain being retained and used for the next analysis. All sequences were aligned using MAFFT v7.471 and trimmed using trimAL v1.4.1 with the thresholds of -gt = 0.9 and -cons = 20. The ML phylogenetic tree was reconstructed by IQ-TREE based on the best substitution model JTT+G+F screened by ProtTest3, and with 2000 bootstrap iterations.

*PHB*, *PHV*, *REV* and *CNA* belong to *C3HDZ* gene family (Prigge et al., 2005). The *A. thaliana* genome contains five *C3HDZ* genes, which were used as queries for BLASTP searches with E-value of less than 1e^-10^ against 49 plant genomes and transcriptomes (**Supplementary Table S1**). After annotation with InterProScan, sequences containing “Homeobox_dom”, “START_dom” and “MEKHLA” domains were retained and used for the next analysis. All sequences were aligned by MAFFT v7.471, and then trimmed them by the trimAL v1.4.1 software under the control of -gt = 0.9 and -cons = 30. The trimmed sequences were used to reconstruct the ML phylogenetic tree using IQ-TREE based on the JTT+I+G+F model selected by ProtTest3 with 2000 bootstrapping events.

*INO* belongs to the *YAB* gene family (Finet et al., 2016). The *A. thaliana* genome contains six *YAB* members, which were used as queries to perform BLASTP searches against 49 plant genomes and transcriptomes (**Supplementary Table S1**) with an E-value threshold of 1e^-10^. The *YAB* homologs harbor the “YABBY” domain in addition to the C2-C2 zinc finger domain. The reported *YAB* sequence of *Huperzia selago* (Accession number: ASU87387) was collected from its transcriptome sequence (Evkaikina et al., 2017). The sequences were aligned by MAFFT v7.471 and trimmed by trimAL v1.4.1 with -gt = 0.9 and -cons = 20. The ML phylogenetic tree was reconstructed by IQ-TREE with the JTT+G+F model screened by ProtTest3 with 2000 bootstrapping events.

*ATS* belongs to the *KAN* gene family (McAbee et al., 2006). There exist four members in the *A. thaliana* genome, which were used as queries to perform BLASTP searches against 49 plant genomes and transcriptomes (**Supplementary Table S1**) with an E-value of 1e^-23^. The blast hits were annotated with InterProScan, and sequences containing the GARP (GOLDEN2, ARR-B Class, Par1 proteins) domain were retained and used for the next analysis. These sequences were aligned by MAFFT v7.471 and trimmed using trimAL v1.3 with the threshold of -gt = 0.55. The ML phylogenetic tree was reconstructed using IQ-TREE based on the best substitution model JTT+G+F tested by ProtTest3 with 2000 iterations of bootstraps.

*ETT* belongs to the *ARF* gene family (Finet et al., 2013). There exist 23 members in the *A. thaliana* genome, which were used as queries to perform BLASTP searches against 49 plant genomes and transcriptomes (**Supplementary Table S1**) with an E-value threshold of 1e^-40^. Sequences containing “B3”, “ARF” and “AUX/IAA” domains were designated as candidate *ARF*-class homologs. The AUX/IAA proteins contain two conserved C-terminal domains, referred to as III and IV (Finet et al., 2013). The sequences were aligned by MAFFT v7.471 and then trimmed using trimAL v1.4.1 with the thresholds of -gt = 0.9 and -cons = 20. The ML phylogenetic tree was reconstructed using IQ-TREE based on the JTT+G+F model selected by ProtTest3 with 2000 iterations.

### Identification of lineage-specific domains/motifs

The lineage-specific domains/motifs contained in the identified proteins were predicted using the online MEME program (Bailey et al., 2015) (https://meme-suite.org/meme/tools/meme) with the parameter settings as follows: the occurrence rate of a single motif was no greater than one per sequence; the motif width was between 6 and 50 amino acids; the maximum number of identified motifs was 25. Other parameters were set to default values. The identified domains/motifs and their positions within the amino acid sequence were then mapped to known conserved domains, those without homology to the conserved domains were identified as lineage-specific or unique.

### Substitution rate test between gene lineages

The CodeML program implemented in the PAML v4.8 package was used to test shifts in substitution rates between specified foreground and background branches (Yang, 1997). Likelihood Ratio Tests (LRTs) were used to compare the one ratio model that assumes a constant ω (dN/dS = non-synonymous/synonymous substitutions) along all tree branches (ω0) against the two-ratio model that assumes a different ratio for the designated foreground branch (ωf) relative to the remaining background branches (ωb). The *ANT* clade in the *AP2/ERF* gene family, the clade C in the *CUC* family, the clade C in the *BLH* family, the *SPL* clade in the *SPEAR* family, the clades C and D in the *C3HDZ* family, the *INO* clade in the *YAB* family, the *KAN* and *ATS* clades in the *KAN* family, and the *ETT* clade in the *ARF* gene family were designated as foreground branches, respectively. A chi-squared distribution was assumed for 2*Δℓ* with the difference between np2 and np1 as the degree of freedom (difference between the parameter number of the one ratio and the two-ratio models) (Jeffares et al., 2015).

### Comparative analysis of gene expression across organs and species

The gene expression data of *Physcomitrella patens* (E-MTAB-3069) (http://www.ebi.ac.uk/arrayexpress) (Ortiz-Ramirez et al., 2016), *Adiantum capillus* (PRJNA593361) (https://www.ncbi.nlm.nih.gov/sra) (Fang et al., 2021), *Ginkgo biloba* (T0001) (https://ginkgo.zju.edu.cn/project/T0001) (Bai et al., 2022), and rice (PRJNA591969) (https://www.ncbi.nlm.nih.gov/sra) (Zhao et al., 2020) were collected to investigate the spectrum of expression variation of key integument regulatory genes in various organs across species. The reference genome of *A. capillus* was downloaded from the NCBI database (https://www.ncbi.nlm.nih.gov/bioproject) under the BioProject accession number PRJNA593372 (Fang et al., 2021). Clean reads were mapped to their respective reference genomes using HISAT2 (Kim et al., 2019). Gene expression levels were quantified by TPM (transcripts per million) and calculated using the prepDE.py script implemented in the StringTie package (Pertea et al., 2015), and then presented with heatmaps plotted by the R package pheatmap (version 1.0.12).

## Results

### Origin and evolution of genes involved in integument development at the stage of ovule primordium initiation

A total of 361 *ANT* homologs were identified from 49 plant genomes and transcriptomes (**Supplementary Table S2**). Seven sequences were excluded from the phylogenetic analyses for they could not be aligned properly. The phylogenetic tree showed that 354 *ANT* homologs divided into two main groups: the *basalANT* group which was further split into clade A (*ERF*) and clade B (*WRI1* and *WRI3*/*4*), and the *euANT* group consisting of clade C (*AIL2*, *AIL3*/*4*, *AIL5*, and *AIL6*/*7*) and clade D (including *ANT* and *AIL1*) (**Supplementary Figure S1-1**). The homologous sequences of both the *basalANT* and *euANT* groups can be traced back to bryophyte genomes. However, the *ANT* orthologs were only present in angiosperm plants, which originated *via* gene duplications and subsequent diversification of an ancestral gymnosperm gene. Comparing gene structure between *ANT* orthologs and other genes revealed two additional motifs (motif 14 and 19) in the *ANT* orthologs (**Supplementary Figures S1-1**, **S2-1**). Substitution rate tests revealed the significant rate difference between *ANT* and other lineages (*p* < 0.05) (**Supplementary Table S3**).

A total of 269 *CUC* homologs were identified and used to reconstruct the phylogenetic tree (**Supplementary Table S2**). The tree showed that the *CUC* homologs fell into three main clades: clade A, clade B, and clade C (**Supplementary Figure S1-2**). The *CUC* homologous sequences can be traced back to the origin of terrestrial plants. However, the *CUC1*/*2*/*3* orthologs were only present in angiosperm plants, which originated *via* angiosperm-specific gene duplication events. Comparing gene structures between different *CUC* homologs revealed one additional motif (motif 18) in *CUC1*/*2* orthologs (**Supplementary Figures S1-2**, **S2-2**). Substitution rate tests did not show rate differences between different lineages (**Supplementary Table S3**).

### Origin and evolution of genes involved in integument development at the stage of ovule patterning

A total of 363 *BLH* homologs were identified from 49 plant genomes and transcriptomes (**Supplementary Table S2**) and used to reconstruct the phylogenetic tree (**Supplementary Figure S1-3**). The tree showed that the *BLH* homologs could be divided into three main clades: clade A, clade B, and clade C (**Supplementary Figure S1-3**). The homologous sequences of *BLH* were found in bryophytes, but the *BEL1* orthologs were only present in angiosperm plants. Compared to its ancestral sequence, *BEL1* orthologs underwent both motifs gain and loss events (**Supplementary Figures S1-3**, **S2-3**). Substitution rate tests revealed a significant difference between *BEL1* and other lineages (*p* < 0.01) (**Supplementary Table S3**).

A total of 128 *SPL*/*NZZ* homologs were identified from 49 representative plant genomes and transcriptomes (**Supplementary Table S2**). Eight sequences were excluded from phylogenetic analyses for they could not be aligned properly. The phylogenetic tree displayed that 120 *SPL*/*NZZ* homologs split into four major clades: clade A (*gymnoSPEAR*), clade B (*SPEAR2*/*4*), clade C (*SPEAR1*/*3*), and clade D (*SPL*) (**Supplementary Figure S1-4**). The homologous sequences of *SPL*/*NZZ* have been present in mosses, with the *SPL*/*NZZ* orthologs being only present in angiosperm plants. Compared to its paralogs, *SPL*/*NZZ* lineages lost several motifs following duplication (**Supplementary Figures S1-4**, **S2-4**). Substitution rate tests revealed a significant difference between *SPL*/*NZZ* and other lineages (*p* < 0.05) (**Supplementary Table S3**).

### Origin and evolution of genes involved in the integument development at the stage of ovule morphogenesis

A total of 215 *C3HDZ* homologs were identified from 49 plant genomes and transcriptomes examined (**Supplementary Table S2**) and used to reconstruct the phylogenetic tree (**Supplementary Figure S1-5**). The tree clear indicated that the *C3HDZ* homologs fell into four main clades: clade A (*HDZ1*), clade B (*HDZ2*), clade C (including *CNA*, and *HB8*), and clade D [(*PHX* including *PHB* and *PHV*), and *REV*] (**Supplementary Figure S1-5**). The orthologs of *PHB*, *PHV* and *REV* were angiosperm-specific, but their homologs were already present in charophytes. The homologs *HDZ1* and *HDZ2* were restrictedly present in non-flowering vascular plants and have lost in angiosperms. The genes of the *C3HDZ* family were highly conserved without structural variation between different members (**Supplementary Figures S1-5**, **S2-5**). Substitution rate tests revealed a significant difference between *C3HDZ* and other lineages (*p* < 0.01) (**Supplementary Table S3**).

**Figure 2:**
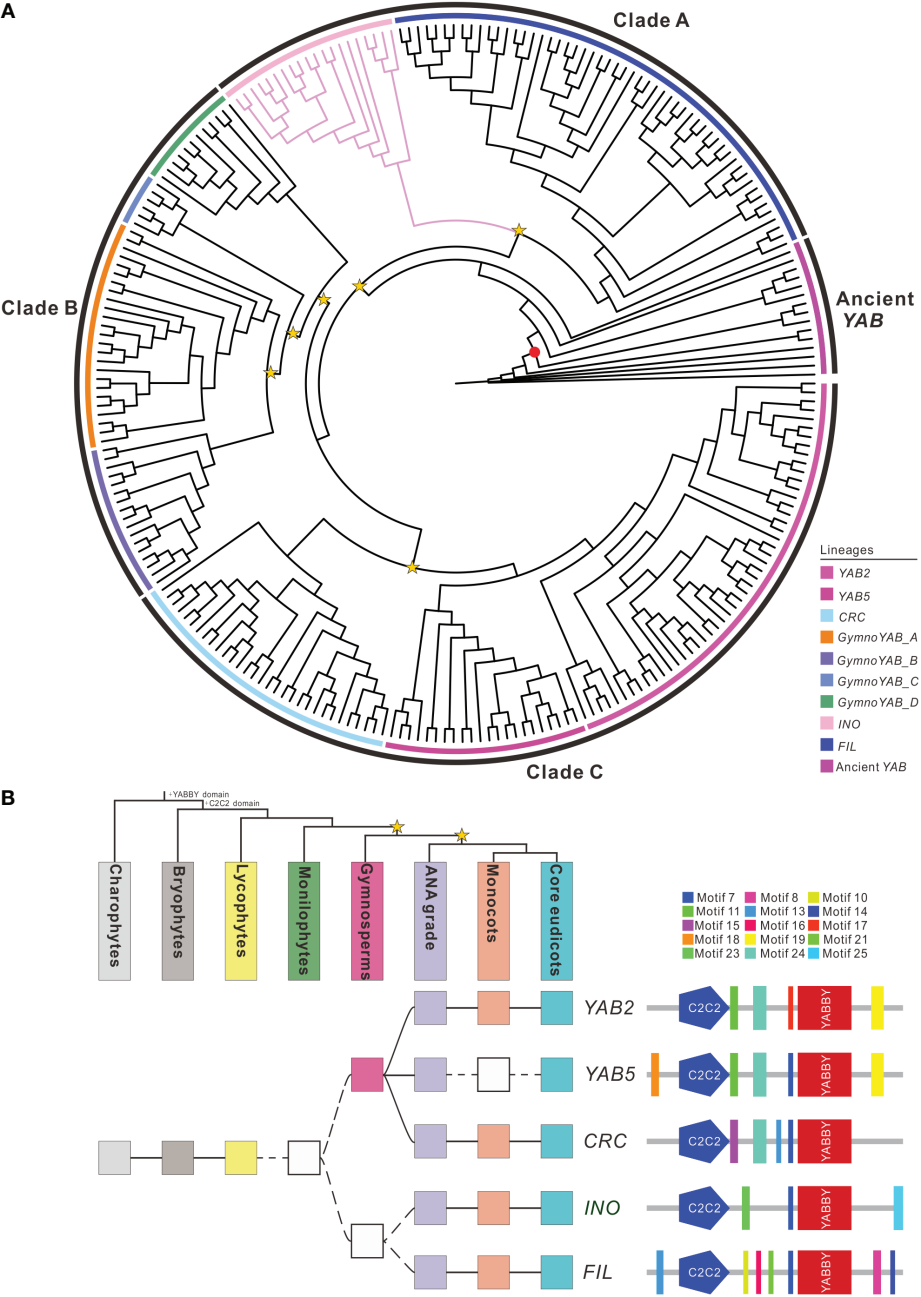
Phylogeny and domain architecture of the *YABBY* homologs. **(A)** Phylogenetic tree of 251 *YABBY* homologs identified from diverse green plants. The red dot indicates the branch support values of BP > 85, while the yellow star indicates the large-scale duplication events. Branches marked with pink indicate the *INO* orthologs. The outer black circles represent the range of different clades and the inner colored circles indicate sublineages within each clade. The support values for each node are shown in **Supplementary Figure S1-6**. **(B)** Duplication history and domain architecture of the *YABBY* homologs. Filled squares indicate the presence of the corresponding members, open squares indicate lack of data. The color of squares is corresponding to the top organismal tree. The yellow stars in the tree indicate whole-genome duplication events. The diagram on the right demonstrates the domain/motif composition of different duplicates. The known conserved domains/motifs included: C2-C2 zinc finger (C2C2) and the YABBY domain. The unnamed domains/motifs are linage-specific and are marked with the colors corresponding to the sequence logos in **Supplementary Figure S2-6**.

A total of 251 *YAB* homologs were identified from 49 representative plant genomes and transcriptomes examined (**Supplementary Table S2** and **Supplementary Figure S1-6**) and used to reconstruct the phylogenetic tree (**Figure 2A**). The *YAB* homologs formed three major clades in the tree: clade A (including *FIL* and *INO* lineages), clade B (*YAB* homologs from gymnosperms) and clade C (including *YAB2*, *YAB5*, and *CRC* lineages) (**Figure 2A**). The homologous sequences of *YAB* were present in chlorophytes, mosses and lycophytes, but were absent in monilophytes. The *INO* orthologs were only present in angiosperm plants and had two additional motifs (motif 23 and 25) compared to its paralogs (**Figure 2B** and **Supplementary Figure S2-6**). Substitution rate tests revealed a significant rate difference between *INO* and other lineages (*p* < 0.01) (**Supplementary Table S3**).

A total of 199 *KAN* homologs were identified from 49 plant genomes and transcriptomes (**Supplementary Table S2**) and used subsequently to reconstruct the phylogenetic tree (**Supplementary Figure S1-7**). The *KAN* homologs clustered into three main clades: clade A (including *gymnoKAN_A* and *gymnoKAN_B* lineages), clade B (*ATS* lineage), and clade C (including *KAN1* and *KAN2*/*3* lineages) (**Supplementary Figure S1-7**). The presence of the *KAN* homologous sequences was limited to land plants, suggesting an origin of this gene family along with the invasion of the land by plants. However, the *ATS* orthologs were only present in angiosperms and lost several motifs following duplication compared to its paralogs (**Supplementary Figures S1-7**, **S2-7**). Substitution rate tests displayed a significant rate difference between *ATS* and other lineages (*p* < 0.01) (**Supplementary Table S3**).

A total of 685 *ARF* homologs were identified (**Supplementary Table S2**) and used subsequently to reconstruct the phylogenetic tree. In the tree, the *ARF* homologs were grouped into three major clades: clade A (including *ARF1*, *ARF2*, *ETT*/*ARF4*, and *ARF9*), clade B (including *ARF5*/*7* and *ARF6*/*8*), and clade C (including *ARF10*/*16* and *ARF17*) (**Supplementary Figure S1-8**). The *ARF* homologous sequences could be traced back to charophytes, suggesting an early origin of this gene family. The *ETT* orthologs were only present in angiosperm plants, and lost the Aux/IAA dimerization domain and several motifs following duplication (**Supplementary Figures S1-8**, **S2-8**). Substitution rate tests revealed a significant rate difference between *ETT* and other lineages (*p* < 0.01) (**Supplementary Table S3**), possibly due to repeated domain/motif losses.

### Expression and function of key genes related to integument development in different plants

**Figure 3:**
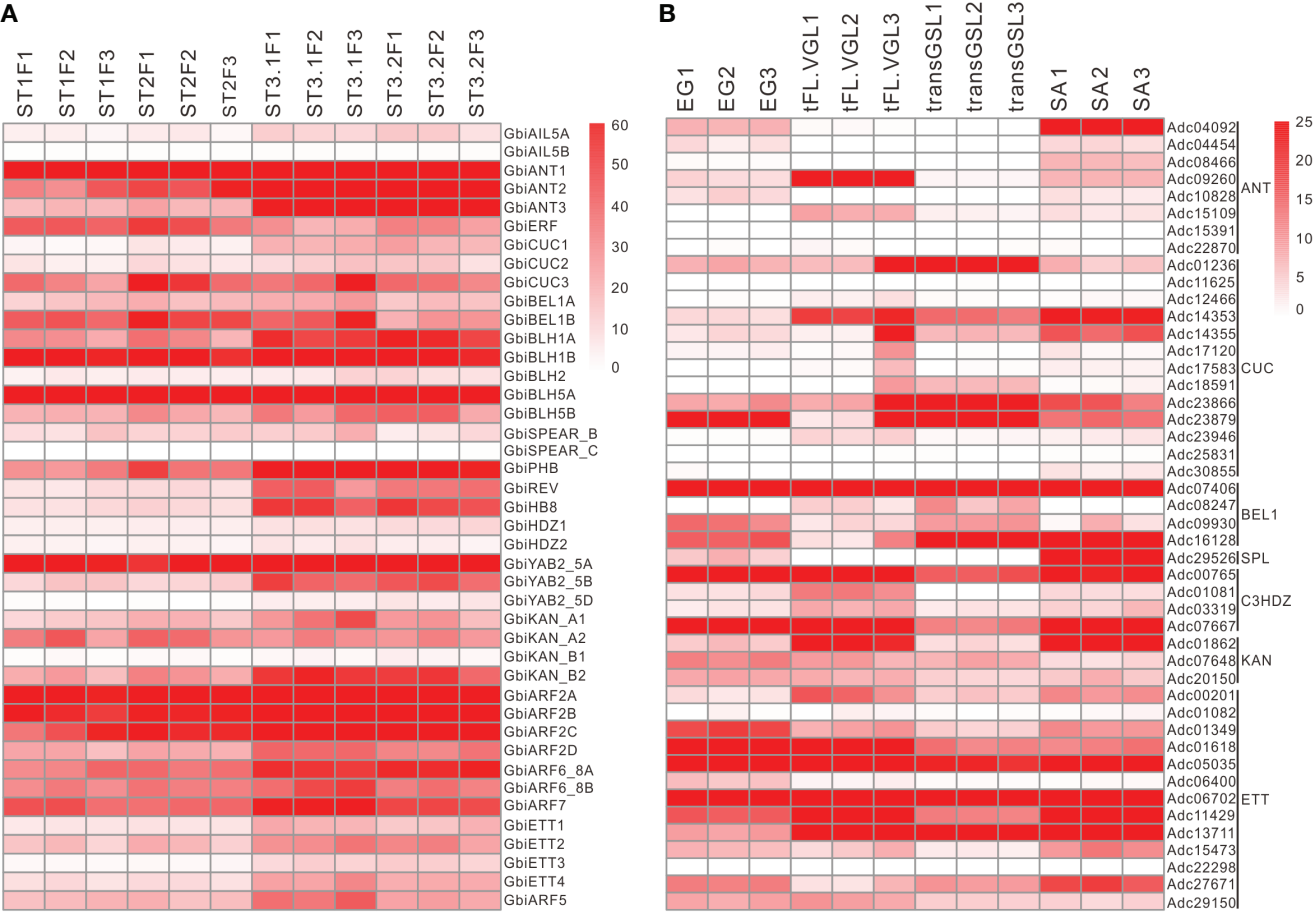
Expression of key genes related to integument development in *Ginkgo biloba* **(A)** and *Adiantum capillus* **(B)**. The four developmental stages of *G. biloba* included the vegetative (ST1), the initial differentiation (ST2), the early exuberant differentiation (ST3-1) and the late exuberant differentiation (ST3-2) stages. The four tissues of *A. capillus* included the embryo gametophyte (EG), the transFL vegetative growth leaf (tFL-VGL), the transGSL green sporangium leaf (transGSL) and the stem apical (SA). Each stage or tissue contained three independent biological replicates.

Tracking the expression pattern of key genes associated with integument development in various organs and plants showed that the homologs of key integument regulatory genes were not only expressed in the ovules of *G. biloba* and rice but also in the leaf-like organs of *A. capillus*, even in *P. patens* tissues (**Figure 3** and **Supplementary Figure S3**). For instance, the *CUC*, *BEL1*, *C3HDZ*, *KAN*, and *ETT* orthologs in *A. capillus* were highly expressed in the gametophyte with embryo (EG), the vegetative growth leaf (VGL), the leaf bearing green sporangium (GSL), the leaf bearing juvenile sporangium (JSL), the leaf bearing mature sporangium MSL), and the leaf bearing dehiscent sporangium (DSL) (**Figure 3** and **Supplementary Figure S3**). Moreover, *ANT*, *CUC*, *C3HDZ*, *KAN*, and *ETT* orthologs of *P. patens* have high expression in the protonema and gametophyte tissues (**Supplementary Figure S3**). These results indicated that the developmental regulatory program composed by the co-expression of these genes was not unique to the integument, but was common to different leaf-lateral organs, and had been formed prior to seed-formation.

## Discussions

### Evolution of the core regulatory genes associated with integument development

Reconstruction of gene phylogenies revealed that all the genes of interest have experienced duplication-divergence events in their evolutionary history. The ancestral genes of *ANT*, *BEL1*, *C3HDZ* and *ETT* have been duplicated in bryophyte and/or seedless vascular plant genomes, prior to the origin of seed plants (**Supplementary Figure S1** and **Table 1**). In contrast, the ancestor genes of *CUC*, *SPL*, *INO* and *ATS* were duplicated along with the origin of gymnosperms, followed by another round of duplication in angiosperm genomes (**Supplementary Figure S1**). *INO* is usually recognized to be angiosperm-specific, which originated from the duplication and diversification of the *YAB* ancestor in flowering plant genomes without orthologs in non-flowering plants. Strictly speaking, however, the other genes including *ANT*, *CUC*, *BEL1*, *SPL*, *ATS* and *ETT*, were also angiosperm-specific because they were all derived from the angiosperm-specific duplication events. Subsequent diversification in gene structure and expression patterns have been detected between these genes and their paralogs in angiosperm genomes, as well as their gymnosperm homologs (co-orthologs) that predate those duplications (**Table 1**). For instance, *AIL1* has orthologs in gymnosperm genomes. It duplicated in the ancestor of angiosperms followed by diverging in gene structure and functions between paralogs, and finally gave rise to *ANT* (**Supplementary Figure S1-1**). Unlike other genes, the paralogs of the *C3HDZ* genes, *PHB*, *REV* and *CNA*, were relatively conserved in gene structure, maintaining the same motif pattern after duplication (**Supplementary Figure S1-5** and **Table 1**). Subfunctionalization of these paralogs most likely resulted from variations of the regulatory motif in the noncoding region. The results of this study thus not only demonstrate that different core regulatory genes involved in integument development which are normally considered typical or even unique in seed plants have actually homologs in seedless plants, but also indicate that gene duplication followed by subfunctionalization of duplicates play a decisive role in the origin and evolution of the integument development genes.

**Table 1:** Summary table showing the characteristics of the genes associated with integument development.

Ovule development stage	Gene*	First occurrence of homologues	Gymnosperm homologues	Characteristic domains	Motif gain/loss
Ovule primordia initiation	*ANT*	Bryophytes	*ANT*/*AIL1*	R1 R2	Motifs 14 and 19 gain
	*CUC1*/*2*/*3*	Bryophytes	*CUC1*/*2*/*3*	NAC (A, B, C, D and E)	Motif 18 gain
Ovule patterning	*BEL1*	Bryophytes	*BEL1*/*BLH2*/*4*	SKY BEL-B HD	Motif 21 gain and motifs 13/14 loss
	*SPL*	Bryophytes	*SPL*	SPL EAR	Motifs 16, 19, 20 and 25 loss
Ovule morphogenesis	*PHV*/*PHB*/*REV*	Charophytes	*PHX*/*REV*	HD START MEKHLA	Conserved
	*INO*	Charophytes	*INO*/*FIL*	C2C2 YABBY	Motifs 23 and 25 gain
	*ATS*	Bryophytes	*ATS*	GARP	Motifs 5, 7, 8 and 10 loss
	*ETT*	Charophytes	*ETT*/*ARF4*	B3 ARF AUX/IAA	Motifs 11, 18, III and IV loss

*Refer to **Supplementary Figures S1**, **S2** for detailed information for each gene.

### Expression and function of the core integument developmental genes prior to seed-formation

*ANT*, *CUC*, *BEL1*, *SPL*, *C3HDZ*, *INO*, *ATS* and *ETT* function as master regulators of integument development in angiosperms. Their homologs, however, play varied roles in seedless plants. The *ANT*/*AIL* homologs have been found to be expressed in the emerging gametophore apical cells, which was in accordance with our study (**Supplementary Figure S3-3**), in *P. patens* to determine stem cell identity (Aoyama et al., 2012). They were also expressed in the emerging fertile fronds in *Ceratopteris richardii* (Bui et al., 2017) and *A. capillus* (**Figure 3** and **Supplementary Figure S3-2**). Horst et al. reported that the *BEL1* homolog functioned as a master regulator for the gametophyte-to-sporophyte transition in *P. patens*; loss of function of *PpBELL1* generated bigger egg cells unable to form embryos (Horst et al., 2016). The *SPL*/*NZZ* homolog has been shown to act as a transcription repressor to regulate auxin homeostasis across embryophytes and participate in controlling sporogenesis (Chen et al., 2014; Wei et al., 2015) and lateral organ morphogenesis (Li et al., 2008). Duplication and neofunctionalization of the *C3HDZ* genes have been revealed to occur in the ancestor of euphyllophytes, with a functional shift from regulating sporangium development to initiate of lateral primordia and leaf development (Vasco et al., 2016). The identification of an *INO*/*YAB* homolog in *H. selago* (Lycopodiales) suggests that the ancestral *INO*/*YAB* gene has evolved in the common ancestor of the vascular plants to control leaf formation (Evkaikina et al., 2017). The *ATS*/*KAN* homologs seem to be involved not only in establishing leaf polarity in *Selaginella moellendorffii* but also in the initiation of sporangium development (Zumajo-Cardona et al., 2019). As one of the core components of the nuclear auxin pathway, the *ETT*/*ARF* homologs have participated in regulating many aspects of plant growth and development since the early stages of green plant evolution by regulating transcription of auxin responsive genes (Kato et al., 2018; Martin-Arevalillo et al., 2019). The most notable is that these distinct transcription factors have assembled and acted cooperatively to form a genetic framework controlling the initiation and patterning of leaves during the origin and early evolution of euphyllophytes (Fang et al., 2021). The framework seems to have maintained throughout the development of leaf-like lateral organs in ferns and seed plants.

### The nature and origin of integuments from an evo-devo perspective

Did the integument arise *de novo*, or evolve from elaboration of pre-existing structures? Providing a reasonable answer to this question depends largely on the clarification of the fundamental nature of integuments because there is little consensus between these two hypotheses in regards to the genetic basis of origin and the evolutionary route.

Gene expression analyses have clearly shown the expression of the *ANT*/*AIL* homologs in developing integuments in *G. biloba* (Wang et al., 2016; Zumajo-Cardona et al., 2021; D'Apice et al., 2022), the conifer *Pinus thunbergii* (Shigyo and Ito, 2004; Yamada et al., 2008) and different species of *Gnetum* (Yamada et al., 2008; Zumajo-Cardona and Ambrose, 2021). Larsson et al. and Schlögl et al. showed the roles of the *CUC* homologs in regulating the differentiation of the shoot apical meristem (SAM) and embryogenesis in *Picea abies* (Larsson et al., 2012) and *Araucaria angustifolia* (Schlogl et al., 2012), respectively, as consistent with the roles in *Arabidopsis* (Chakraborty and Roy, 2019). A strong expression of the *BEL1* homolog has been detected in the integument in *G. biloba* (Zumajo-Cardona et al., 2021; D'Apice et al., 2022). The homologs of *C3HDZ* and *ATS*/*KAN* were expressed throughout ovule development in *G. biloba* (Zumajo-Cardona et al., 2021; D'Apice et al., 2022) and the *Gnetum* species (Zumajo-Cardona and Ambrose, 2021). The *INO*/*YAB* homologs were also found to be expressed in the ovule integument in *Ephedra distachya* (Finet et al., 2016). Functional characterization of these genes in model plants has revealed that they are mostly the key components of the genetic program that patterns the laminar structure of leaf-like lateral organs (Mathews and Kramer, 2012). These genes participate in the establishment of abaxial-adaxial polarity during the development of integuments and other leaf-like lateral organs. Not only at the molecular level, integuments and leaves also share a number of defining features at the morphological level, including similar modes of organ initiation, determinant growth, and bilateral symmetry. Given the shared features of patterning and morphogenesis, serial homology has been postulated between leaves and integuments (Kelley and Gasser, 2009). Serial homology denotes the similarity of repetitive structures within the same organism (within-individual homology) (Minelli and Fusco, 2013), which is distinct from the historical (phylogenetic) homology that means the similarity of structures in two or more taxa descended from a common ancestor (between-species homology) (Mabee et al., 2020). The idea of serial homology is often rejected by traditional comparative biologists because for them homology is about the comparison of different species (Brigandt, 2003). However, serial homology is widely accepted and utilized in evolutionary developmental biology, which intends to account for the origin of similar structures both within and between organisms and for structural identity in ontogeny and phylogeny (Brigandt, 2003; DiFrisco et al., 2022; Fusco, 2022).

Two structures in the same organism are serial homologs if they share a large proportion of their genetic architecture and developmental pathways. The sharing of core regulatory genes supports the inference of integuments as leaf serial homologues. The results of this study demonstrate that the genetic regulatory network composed of the shared genes had been assembled at the early stage of euphyllophyte evolution, patterning a leaf by establishing polarities prior to the origin of seed plants. The underlying mechanism is reused in integument patterning while seed formation. The integument is thus unlikely to arise *de novo* from the perspective of developmental regulation, but evolved from the modification of the pre-existing genetic regulatory apparatus. It could be argued that the reuse or sharing of the leaf patterning gene network in integument development does not necessarily indicate a duplication of serial homologs. The genetic overlap may also be due to co-option and deep homology. Both terms are commonly used to describe the repeated use of the same genes or gene networks even in phylogenetically distant lineages, without specification of the implications for the corresponding phenotype (DiFrisco et al., 2022). Deep homology is often seen as an evolutionary residue of co-option events, with the shared genes or conserved gene networks being convergently recruited into different developmental processes to build morphologically and phylogenetically disparate features (Shubin et al., 1997; DiFrisco et al., 2022). The resulting morphological structures are usually not thought to be homologous (Tschopp and Tabin, 2017). The sharing of the leaf patterning gene network in integument translates to acquisition of the laminar organ identity at different stem segments within the same individual organism, strongly suggesting the origin of the integument *via* reusing and subsequent individuation of serially homologous structures but not due to co-option or ordinary gene sharing and pleiotropy which is often referred to as homocracy, i.e. shared patterns of regulatory gene expression among organs (Nielsen and Martinez, 2003). The recognition of ovules as meristematic axes provides further support for the stem segment-specific modification hypothesis of integument origin. Gross-Hardt et al. showed that the meristematic gene *WUSCHEL* (*WUS*) was expressed in the nucellus to regulate integument initiation in the chalaza by generating a downstream signal (Gross-Hardt et al., 2002). Just like the formation of leaf primordia in apical meristems, integuments arise from the nucellar meristem as novel lateral organs (Mathews and Kramer, 2012). This view is consistent with the axial theory proposed by several botanists in the nineteenth century that the nucellus is of the nature of a bud bearing the integuments as lateral foliar appendages (Worsdell, 1904).

The master ‘switches’ trigging the modification of the genetic program to specify the integument identity and development during the emergence of gymnosperms remain unclear. Identification of the upstream “selector” genes that are tightly linked to the identity of integument and contribute to distinct character development relative to other lateral organs is crucial for evaluating the serial homology hypothesis (DiFrisco et al., 2022). Duplication and subfunctionalization of known development regulatory genes might also facilitate the evolution of the regulatory network in tissue-developmental stage specificity. For instance, *AIL* is a pleiotropic gene which is involved not only in initiation and development of integument but also in initiation and growth of all plant organs except roots through control of cell proliferation (Horstman et al., 2014). This pleiotropic effect is the result of its control of cell proliferation during organogenesis, which seems to be a conserved function in the entire core eudicotyledons. The specialization of the descendant paralogous is not entirely dependent on changes to *cis*-regulatory DNA because there is growing evidence that changes to the coding regions of transcription factors play a much larger role in the evolution of developmental gene regulatory networks than originally imagined (Jarvela and Hinman, 2015). After duplication, relaxed constraints allow paralogous genes to diverge through mutations or exon shuffling to gain or loss motifs/domains. Just as cis-regulatory changes avoid pleiotropy by modulating the binding site composition, the changes in coding region can also lead functional specialization of paralogs by affecting alternative splicing, post-translational modification and protein-protein interaction. Nearly all the genes surveyed in this study exhibited structural variations in the coding regions, except *C3HDZ* (**Table 1** and **Supplementary Figure S1-5**). Of them, *ANT* acquired two additional motifs following duplication (**Supplementary Figure S1-1**), while *CUC*, *SPL*, *ATS* and *ETT* lost motifs compared to their paralogs (**Supplementary Figure S1**). *BEL1* and *INO* have undergone both motifs gain and loss events (**Figure 2B** and **Supplementary Figure S1**). Meister et al. (2005) and Gallagher and Gasser (2008) have shown that the C-terminal regions of INO which contain the novel motifs acquired following duplication were essential for its function in controlling the initiation and asymmetric growth of the outer integument by interacting with SUP (Meister et al., 2005; Gallagher and Gasser, 2008).

**Figure 4:**
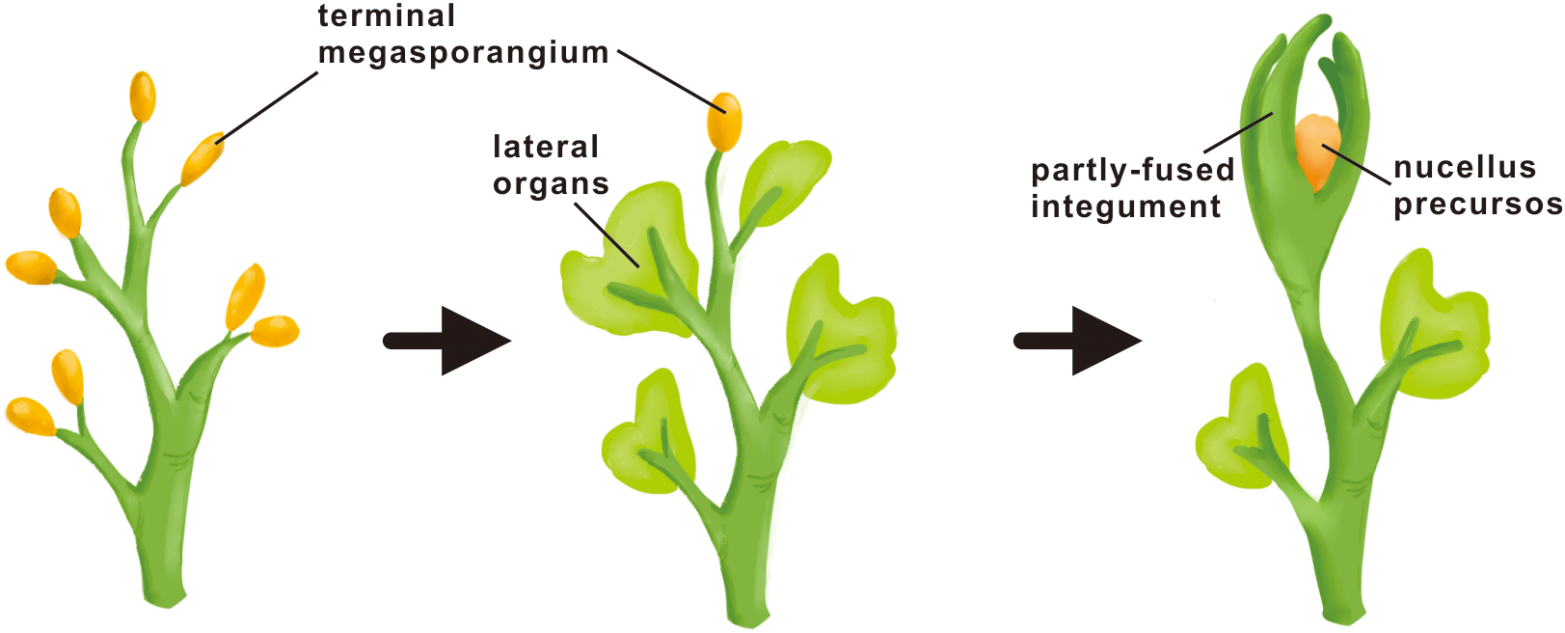
Hypothetical mode of integument origin. The origin of the integument is neither a direct transformation process nor a *de novo* process, but a successive transformation process. In early plant evolution, the sterilized telomes (branches) were first transformed into leaf-like lateral organs in seedless plants, and then the leaf-like lateral organs were further transformed into the integument at the time of seed occurs.

The extant integuments are morphologically quite distinct from that of fossil plants, making it difficult to decide whether they are historically homologous structures orchestrated by the same developmental system. There exist two alternative perspectives. The morphological distinctions possibly reflect the difference in their derivation with the lobed structure surrounding ovules in ancestral seed plants derived from transformation of sterile branches or sterilized sporangia, as predicted by the telome theory. The other perspective is that the envelopes encircling nucellus in the ovules of both extinct and extant taxa are historically (phylogenetically) homologous, possessing the common nature as lateral organs but undergoing differing degrees of evolutionary modification. It is impossible to examine how development of the ancestral structure might have been regulated. However, the latest research on the most primitive *Genomosperma* ovules showed that the organization of the lobed integument was highly plastic, with the lobe number and the degree of fusion varying considerably among individual fossils, exhibiting strong ontogenic (developmental) and polymorphic signals (Meade et al., 2021). Considering their findings above, the authors speculated that the lobes of the *Genomosperma* integument developed similarly to a whorl of extant floral organs and have been regulated by similar mechanisms to extant seed plant reproductive organs (Meade et al., 2021). The results of evolutionary and functional analysis of multiple integument regulatory genes seem to support their speculations. The ancestral integument structure and extant integuments, different though they seem, might share a gene network that formed *via* the stem segment-specific modification of the pre-existing genetic program for other lateral organs. The integument very likely evolved as a consequence of successive transformations from the modification of terminal branches (telomes) to leaf-like lateral organs and then into integuments (**Figure 4**).

## Conclusions

The origin of evolutionary novelty and the mechanics of innovation are central topics in evolutionary developmental biology. However, the definition of novelty and its relationship to homology are not well defined. If a new morphological trait has been shown to originate through the use and modification of pre-existing gene regulatory networks, it becomes unclear whether the trait is truly novel or merely modified serial homolog. Given the sharing of a homologous core set of genes, the integument was postulated to be a serially homologous structure in this study, but not arise *de novo*. From the structural and functional perspective, however, the integument drastically diverged from other lateral foliar appendages, facilitating survive and spread of seed plants. The definition of novelty and homology and the criteria of their application seem to remain controversial across the biological hierarchy. Comparing the character states, development processes and the underlying gene networks of different lateral organs within the context of serial vs historical homology may contribute to a more comprehensive understanding of how organs be transformed into one another during ontogeny and phylogeny, which has implication for our understanding of the key developmental events and regulators leading to the origin and evolution of the integument.

## Data availability statement

The datasets presented in this study can be found in online repositories. The names of the repository/repositories and accession number(s) can be found in the article/**Supplementary Material**.

## Author contributions

JY conceived and designed the work. MJ collected and analyzed the data. JJ, CZ, LL, YW, WZ and ZS participated in data analysis. MJ drafted the manuscript. JY revised the manuscript. All authors read and approved the final manuscript.

## Supplementary material

Supplementary Table 1 49 representative genomes or transcriptomes selected in this study for identifying homologous genes. Species, family and the corresponding database are included.

Supplementary Table 2 List of sequences included in the phylogenetic analyses for the *ANT*, *CUC*, *BEL1*, *SPL*, *C3HDZ*, *YABBY*, *KANADI*, and *ARF* gene families, respectively. Species name, gene name, and the accession numbers are included.

Supplementary Table 3 The likelihood ratio tests are performed for the eight gene families investigated in the study. The *ANT* clade in the *AP2/ERF* gene family, the clade C in the *CUC* family, the clade C in the *BLH* family, the *SPL* clade in the *SPEAR* family, the clades C and D in the *C3HDZ* family, the *INO* clade in the *YAB* family, the *KAN* and *ATS* clades in the *KAN* family, and the *ETT* clade in the *ARF* gene family were designated as foreground branches, respectively.

Supplementary Figure 1 Phylogeny and domain architecture of different gene families. Support values are shown for nodes. The scale bar indicates the number of changes per site. Different gene lineages are marked with different colors. **(S1-1) Phylogeny and domain architecture of the *ANT* homologs. (A)** Phylogenetic tree of 354 *ANT* homologs identified from diverse land plants. The red dot indicates the branch support value of BP > 85, while the yellow stars indicate the large-scale duplication events. Branches marked with pink indicate the *ANT* orthologs. The outer black circles represent the range of different clades, and the inner colored circles indicate sublineages within each clade. **(B)** Duplication history and domain architecture of the *ANT* homologs. Filled squares indicate the presence of the corresponding members, open squares indicate absence data. The color of squares is corresponding to the top organismal tree. The yellow stars in the tree indicate whole-genome duplication events. The diagram on the right demonstrates the domain/motif composition of different duplicates. The known conserved domains/motifs included: euANT1, euANT2, euANT3, euANT4, the AP2 domain (R1 and R2) and the linker regions (L). The unnamed domains/motifs are linage-specific and are marked with the colors corresponding to the sequence logos in **Supplementary Figure S2-1**. **(C)** Phylogenetic tree of *ANT* homologs with the support values at each node. **(S1-2) Phylogeny and domain architecture of the *CUC* homologs. (A)** Phylogenetic tree of 266 *CUC* homologs identified from diverse land plants. The red dot indicates the branch support value of BP > 85, while the yellow star indicates the large-scale duplication events. Branches marked with purple indicate the *CUC* orthologs. The outer black circles represent the range of different clades and the inner colored circles indicate sublineages within each clade. **(B)** Duplication history and domain architecture of the *CUC* homologs. Filled squares indicate the presence of the corresponding members, open squares indicate lack of data. The color of squares is corresponding to the top organismal tree. The yellow stars in the tree indicate whole-genome duplication events. The diagram on the right demonstrates the domain/motif composition of different duplicates. The known conserved domains/motifs included: five subdomains of the NAC domain A, B, C, D, and E, the V motif (V), the L motif (L) and the W motif (W). The unnamed domains/motifs are linage-specific and are marked with the colors corresponding to the sequence logos in **Supplementary Figure S2-2**. **(C)** Phylogenetic tree of *CUC* homologs with the support values at each node. **(S1-3) Phylogeny and domain architecture of the *BLH* homologs. (A)** Phylogenetic tree of 363 *BLH* homologs identified from diverse land plants. The red dot indicates the branch support values of BP > 85, while the yellow star indicates the large-scale duplication events. Branches marked with pink indicate the *BEL1* orthologs. The outer black circles represent the range of different clades and the inner colored circles indicate sublineages within each clade. **(B)** Duplication history and domain architecture of the *BLH* homologs. Filled squares indicate the presence of the corresponding members, open squares indicate absence data. The color of squares is corresponding to the top organismal tree. The yellow stars in the tree indicate whole-genome duplication events. The diagram on the right demonstrates the domain/motif composition of different duplicates. The known conserved domains/motifs included: BEL-A (SKY), BEL-B, BEL-C, ZIBEL and the homeodomain (HD). The unnamed domains/motifs are linage-specific and are marked with the colors corresponding to the sequence logos in **Supplementary Figure S2-3**. **(C)** Phylogenetic tree of *BLH* homologs with the support values at each node. **(S1-4) Phylogeny and domain architecture of the *SPEAR* homologs. (A)** Phylogenetic tree of 120 *SPEAR* homologs identified from diverse land plants. The red dot indicates the branch support values of BP > 85, while the yellow star indicates the large-scale duplication events. Branches marked with pink indicate the *SPL* orthologs. The outer black circles represent the range of different clades and the inner colored circles indicate sublineages within each clade. **(B)** Duplication history and domain architecture of the *SPEAR* homologs. Filled squares indicate the presence of the corresponding members, open squares indicate absence data. The color of squares is corresponding to the top organismal tree. The yellow stars in the tree indicate whole-genome duplication events. The diagram on the right demonstrates the domain/motif composition of different duplicates. The known conserved domains/motifs included: the nuclear localization signal domain (NLS), the SPL-motif and the EAR motif (LxLxL). The unnamed domains/motifs are linage-specific and are marked with the colors corresponding to the sequence logos in **Supplementary Figure S2-4**. **(C)** Phylogenetic tree of *SPEAR* homologs with the support values at each node. **(S1-5) Phylogeny and domain architecture of the *C3HDZ* homologs. (A)** Phylogenetic tree of 215 *C3HDZ* homologs identified from diverse green plants. The red dot indicates the branch support values of BP > 85, while the yellow star indicates the large-scale duplication events. The outer black circles represent the range of different clades and the inner colored circles indicate sublineages within each clade. All members of *Arabidopsis* (angiosperms) are associated with integument development. **(B)** Duplication history and domain architecture of the *C3HDZ* homologs. Filled squares indicate the presence of the corresponding members, open squares indicate lack of data. The color of squares is corresponding to the top organismal tree. The yellow stars in the tree indicate whole-genome duplication events. The diagram on the right demonstrates the domain/motif composition of different duplicates. The known conserved domains/motifs included: homeodomain (HD), leucine-zipper (LZ), CESVV, START, *miR165/166* complementary site, HD-SAD (HD-START associated domain) and the MEKHLA domain. **(C)** Phylogenetic tree of *C3HDZ* homologs with the support values at each node. **(S1-6) Phylogeny and domain architecture of the *YABBY* homologs. (A)** Phylogenetic tree of 251 *YABBY* homologs identified from diverse green plants. The red dot indicates the branch support values of BP > 85, while the yellow star indicates the large-scale duplication events. Branches marked with pink indicate the *INO* orthologs. The outer black circles represent the range of different clades and the inner colored circles indicate sublineages within each clade. **(B)** Duplication history and domain architecture of the *YABBY* homologs. Filled squares indicate the presence of the corresponding members, open squares indicate lack of data. The color of squares is corresponding to the top organismal tree. The yellow stars in the tree indicate whole-genome duplication events. The diagram on the right demonstrates the domain/motif composition of different duplicates. The known conserved domains/motifs included: C2-C2 zinc finger (C2C2) and the YABBY domain. The unnamed domains/motifs are linage-specific and are marked with the colors corresponding to the sequence logos in **Supplementary Figure S2-6**. **(C)** Phylogenetic tree of *YABBY* homologs with the support values at each node. **(S1-7) Phylogeny and domain architecture of the *KANADI* homologs. (A)** Phylogenetic tree of 199 *KANADI* homologs identified from diverse land plants. The red dot indicates the branch support values of BP > 85, while the yellow star indicates the large-scale duplication events. The outer black circles represent the range of different clades and the inner colored circles indicate sublineages within each clade. All members from *Arabidopsis* (angiosperms) are associated with integument development. **(B)** Duplication history and domain architecture of the *KANADI* homologs. Filled squares indicate the presence of the corresponding members, open squares indicate absence data. The color of squares is corresponding to the top organismal tree. The yellow stars in the tree indicate whole-genome duplication events. The diagram on the right demonstrates the domain/motif composition of different duplicates. The known conserved domains/motifs included: the GARP (GOLDEN2, ARR-B Class, Par1 proteins) domain and the L-rich motif. The unnamed domains/motifs are linage-specific and are marked with the colors corresponding to the sequence logos in **Supplementary Figure S2-7**. **(C)** Phylogenetic tree of *KANADI* homologs with the support values at each node. **(S1-8) Phylogeny and domain architecture of the *ARF* homologs. (A)** Phylogenetic tree of 658 *ARF* homologs identified from diverse green plants. The red dot indicates the branch support values of BP > 85, while the yellow star indicates the large-scale duplication events. Branches marked with blue indicate the *ETT* orthologs. The outer black circles represent the range of different clades and the inner colored circles indicate sublineages within each clade. **(B)** Duplication history and domain architecture of the *ARF* homologs. Filled squares indicate the presence of the corresponding members, open squares indicate lack of data. The color of squares is corresponding to the top organismal tree. The yellow stars in the tree indicate whole-genome duplication events. The diagram on the right demonstrates the domain/motif composition of different duplicates. The known conserved domains/motifs included: the dimerization domain (DD), the B3 DNA binding domain (B3), the ARF and Aux/IAA domain (Motif III and IV are consensus sequences shared by Aux/IAA proteins). The unnamed domains/motifs are linage-specific and are marked with the colors corresponding to the sequence logos in **Supplementary Figure S2-8**. **(C)** Phylogenetic tree of *ARF* homologs with the support values at each node.

Supplementary Figure 2 Sequence logos of the conserved **(A)** and unique **(B)** domains/motifs of different gene families. The height of the letter indicates its relative frequency at the given position (x -axis) in the domain/motif.

Supplementary Figure 3 Expression of key genes related to integument development in rice (S3-1), *Adiantum capillus* (S3-2) and *Physcomitrella patens* (S3-3). The 33 various developmental stages or tissues of *A. capillus* (S3-2) included the embryo gametophyte (EG), gametophyte (GA), the transUL vegetative growth leaf (tUL-VGL), the transLCGS green sporangium leaf (tCGS-GSL), the transLCMS mature sporangium leaf (tCMS-MSL), the transLCJS juvenile sporangium leaf (tCJS-JSL), the transLCDS dehiscent sporangium leaf (tCDS-DSL), the transCL vegetative growth leaf (tCL-VGL), the transFL vegetative growth leaf (tFL-VGL), the AnotUL vegetative growth leaf (AUL-VGL), the AnotFL vegetative growth leaf (AFL-VGL), the AnotCL vegetative growth leaf (ACJS-JSL), the AnotLCGS vegetative growth leaf (ACGS-DSL), the AnotLCTS sporangium leaf (ACTS-SL), the AnotLCMS mature sporangium leaf (ACMS-MSL), the AnotLCDS dehiscent sporangium leaf (ACDS-DSL), the transMS mature sporangium stage (tMS-MSS), the transGS green sporangium stage (tGS-GSS), the transJS juvenile sporangium stage (tJS-JSS), the AnotJS juvenile sporangium stage (AJS-JSS), the AnotGS green sporangium stage (ATS-SS), the AnotMS mature sporangium stage (AMS-MSS), the transMSL mature sporangium leaf (transMSL), the transGSL green sporangium leaf (transGSL), transDSL dehiscent sporangium leaf (transDSL), transJSL juvenile sporangium stage (transJSL), the AnotJSL juvenile sporangium stage (AnotJSL), the AnotGSL green sporangium leaf (AnotGSL), AnotTSL sporangium leaf (AnotTSL), the AnotMSL mature sporangium leaf (AnotMSL), the AnotDSL dehiscent sporangium leaf (AnotDSL), the transYS young sporophyte (transYS) and the stem apical (SA).
