# Supplementary material for: Does integument arise de novo or from pre-existing structures? ── Insights from the key regulatory genes controlling integument development
Source: Front Plant Sci. 2023 Jan 13;13:1078248. doi: 10.3389/fpls.2022.1078248 (PMC9880897; doi:10.3389/fpls.2022.1078248)
Supplement: Supplementary Table 1 — 49 representative genomes or transcriptomes selected in this study for identifying homologous genes. Species, family and the corresponding database are included. [file Table_1.docx]

**Table S1. 49 representative genomes or transcriptomes selected in this study for identifying homologous genes.**

| Group | Order | Species | Abbr. | Database* |
| --- | --- | --- | --- | --- |
| Angiosperm | Brassicales | Arabidopsis thaliana | At | PLAZA |
|  | Malpighiales | Populus trichocarpa | Ptr | PLAZA |
|  | Fabales | Glycine max | Gma | PLAZA |
|  | Rosales | Fragaria vesca | Fve | PLAZA |
|  | Solanales | Solanum lycopersicum | Sly | PLAZA |
|  | Vitales | Vitis vinifera | Vvi | PLAZA |
|  | Gentianales | Coffea canephora | Cca | PLAZA |
|  | Caryophyllales | Beta vulgaris | Bvu | PLAZA |
|  | Proteales | Nelumbo nucifera | Nnu | PLAZA |
|  | Poales | Brachypodium distachyon | Bdi | PLAZA |
|  |  | Oryza sativa | Os | PLAZA |
|  |  | Zea mays | Zma | PLAZA |
|  | Amborellales | Amborella trichopoda | Atr | PLAZA |
|  | Nymphaeales | Euryale ferox | Efe | PLAZA |
| Gymnosperm | Coniferales | Picea abies | Pab | PLAZA |
|  |  | Picea glauca | Pgl | PLAZA |
|  |  | Picea sitchensis | Psi | PLAZA |
|  |  | Thuja plicata | Tpl | Phytozome |
|  | Cupressales | Taxus baccata | Tba | PLAZA |
|  |  | Sequoiadendron giganteum | Sgi | TreeGenes |
|  | Pinales | Pinus pinaster | Ppi | PLAZA |
|  |  | Pinus sylvestris | Psy | PLAZA |
|  |  | Pinus taeda | Pta | PLAZA |
|  |  | Abies alba | Aal | TreeGenes |
|  |  | Pseudotsuga menziesii | Pme | PLAZA |
|  | Gnetales | Gnetum montanum | Gmo | DRYAD |
|  | Welwitschiales | Welwitschia mirabilis | Wmi | DRYAD |
|  | Cycadales | Cycas micholitzii | Cmi | onekp |
|  | Ginkgoales | Ginkgo biloba | Gbi | GigaDB |
| Pteridophyte | Polypodiales | Ceratopteris richardii | Cri | Phytozome |
|  | Salviniales | Azolla filiculoides | Afi | Fernbase |
|  |  | Salvinia cucullata | Scu | Fernbase |
|  | Selaginellales | Selaginella moellendorffii | Smo | PLAZA |
| Bryophyte | Anthocerotales | Anthoceros angustus | Aan | UZH |
|  |  | Anthoceros punctatus | Apu | UZH |
|  |  | Anthoceros agrestis | Aag | UZH |
|  | Funariales | Physcomitrella patens | Ppa | PLAZA |
|  | Sphagnales | Sphagnum fallax | Sfa | Phytozome |
|  |  | Sphagnum magellanicum | Sma | Phytozome |
|  | Pseudoditrichales | Ceratodon purpureus | Cpu | Phytozome |
|  | Marchantiales | Marchantia polymorpha | Mpo | PLAZA |
| Algae | Chlorokybales | Chlorokybus atmophyticus | Cat | CNSA |
|  |  | Coleochaete orbicularis | Cor | TAPscan |
|  | Chlamydomonadales | Chlamydomonas reinhardtii | Cre | PLAZA |
|  | Mamiellales | Micromonas commoda | Mco | PLAZA |
|  |  | Micromonas pusilla | Mpu | PLAZA |
|  |  | Ostreococcus lucimarinus | Olu | Phytozome |
|  | Klebsormidiales | Klebsormidium flaccidum | Kfl | LINK |
|  | Isochrysidales | Emiliania huxleyi | Ehu | PLAZA |

* PLAZA: <https://bioinformatics.psb.ugent.be/plaza/>; Onekp: <http://www.onekp.com/>; Phytozome: <https://phytozome-next.jgi.doe.gov/>; TreeGenes: <https://treegenesdb.org/FTP/Genomes/>; DRYAD: <https://datadryad.org/stash>; GigaDB: <http://gigadb.org/>; Fernbase: <https://www.fernbase.org/>; UZH: <https://www.hornworts.uzh.ch/en.html/>; CNSA: <https://db.cngb.org/cnsa/>; TAPscan: <https://plantcode.cup.uni-freiburg.de/tapscan/>; LINK: <http://www.plantmorphogenesis.bio.titech.ac.jp/~algae_genome_project/klebsormidium/index.html>.
