# Supplementary material for: Does integument arise de novo or from pre-existing structures? ── Insights from the key regulatory genes controlling integument development
Source: Front Plant Sci. 2023 Jan 13;13:1078248. doi: 10.3389/fpls.2022.1078248 (PMC9880897; doi:10.3389/fpls.2022.1078248)
Supplement: Supplementary Table 3 — The likelihood ratio tests are performed for the eight gene families investigated in the study. The ANT clade in the AP2/ERF gene family, the clade C in the CUC family, the clade C in the BLH family, the SPL clade in the SPEAR family, the clades C and D in the C3HDZ family, the INO clade in the YAB family, the KAN and ATS clades in the KAN family, and the ETT clade in the ARF gene family were designated as foreground branches, respectively. [file Table_3.docx]

| Family | Model | ω (dN/dS) | lnL | 2Δℓ | *df* | *p* value |
| --- | --- | --- | --- | --- | --- | --- |
| *ANT* | One Ratio | ω0= 0.22033 | -416588.723226 | 3.942 | 1 | 4.71E-02 |
|  | Two Ratio | ωf= 0.228484  ωb= 0.218347 | -416586.752349 |  |  |  |
| *CUC* | One Ratio | ω0= 0.23911 | -190465.468212 | 3.494 | 1 | 6.16E-02 |
|  | Two Ratio | ωf= 0.24739  ωb= 0.235607 | -190463.721219 |  |  |  |
| *BEL* | One Ratio | ω0= 0.25499 | -185691.9135 | 18.850 | 1 | 1.41E-05 |
|  | Two Ratio | ωf= 0.27534  ωb= 0.24268 | -185682.4885 |  |  |  |
| *SPEAR* | One Ratio | ω0= 0.39503 | -108538.4436 | 6.157 | 1 | 1.31E-02 |
|  | Two Ratio | ωf= 0.44635  ωb= 0.38828 | -108535.3653 |  |  |  |
| *C3HDZ* | One Ratio | ω0= 0.12119 | -221050.6625 | 126.541 | 1 | 2.34E-29 |
|  | Two Ratio | ωf= 0.11215  ωb= 0.146408 | -220987.3923 |  |  |  |
| *YABBY* | One Ratio | ω0= 0.2378 | -91334.20286 | 13.056 | 1 | 3.02E-04 |
|  | Two Ratio | ωf= 0.285231  ωb= 0.232856 | -91327.67501 |  |  |  |
| *KAN* | One Ratio | ω0= 0.32248 | -168950.5247 | 14.669 | 2 | 6.53E-04 |
|  | Two Ratio | ωf1= 0.310425  ωf2= 0.342284  ωb= 0.346604 | -168943.1902 |  |  |  |
| *ARF* | One Ratio | ω0= 0.20486 | -205273.477215 | 154.101 | 1 | 2.20E-35 |
|  | Two Ratio | ωf= 0.296036  ωb= 0.192613 | -205196.426759 |  |  |  |

**Table S3. The likelihood ratio tests are performed for the eight gene families investigated in the study.**
