# Supplementary figures and images for: Does integument arise de novo or from pre-existing structures? ── Insights from the key regulatory genes controlling integument development
Source: Front Plant Sci. 2023 Jan 13;13:1078248. doi: 10.3389/fpls.2022.1078248 (PMC9880897; doi:10.3389/fpls.2022.1078248)

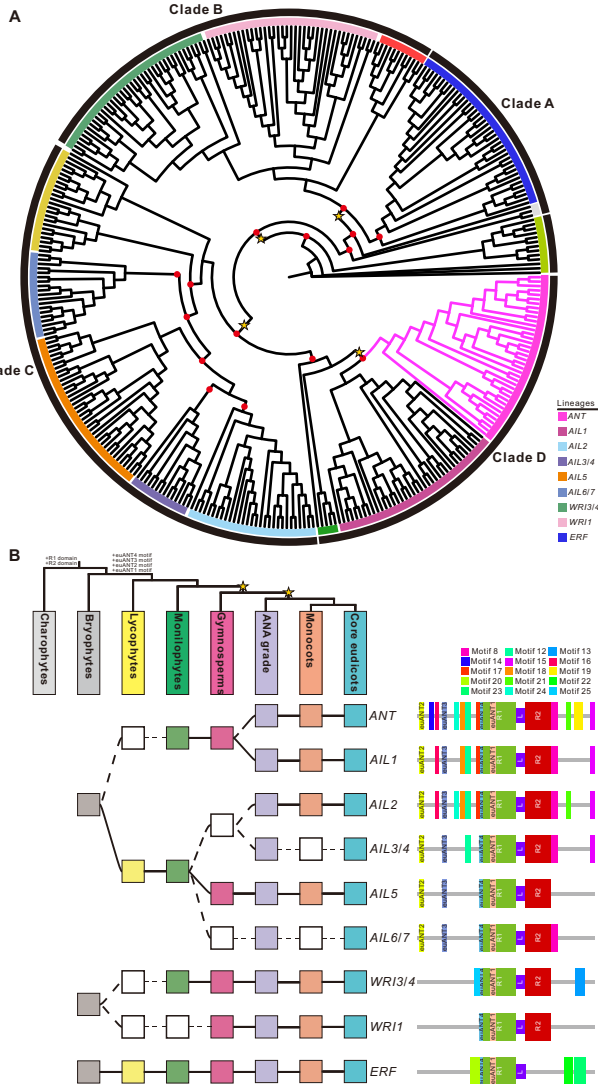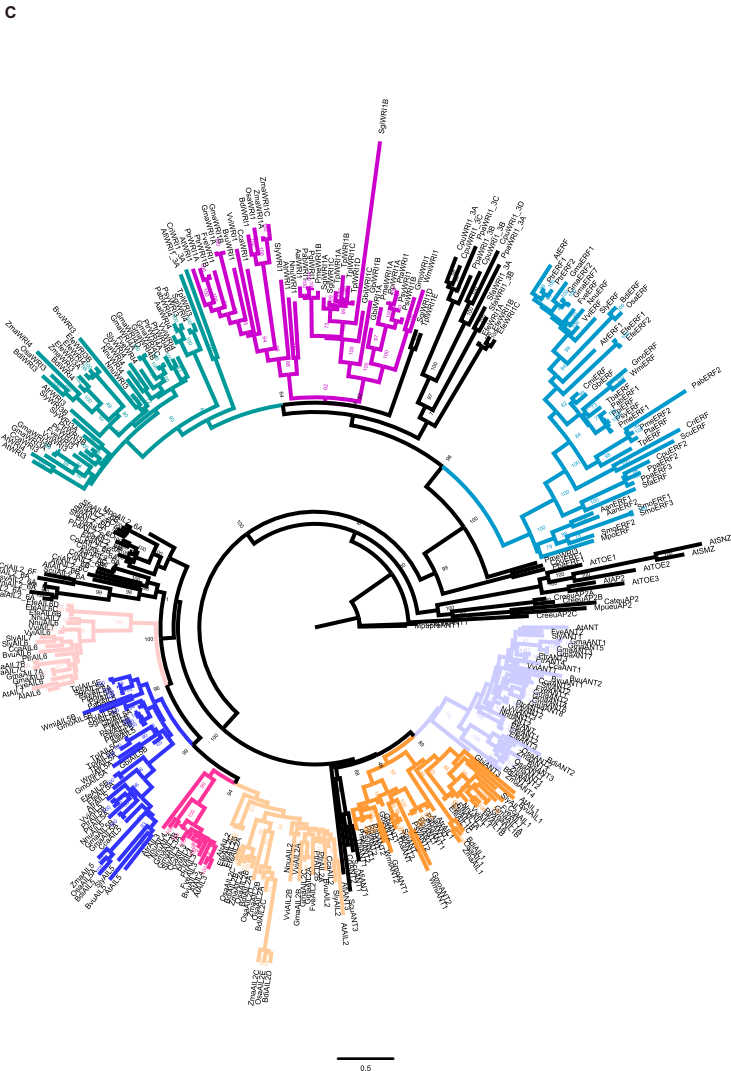

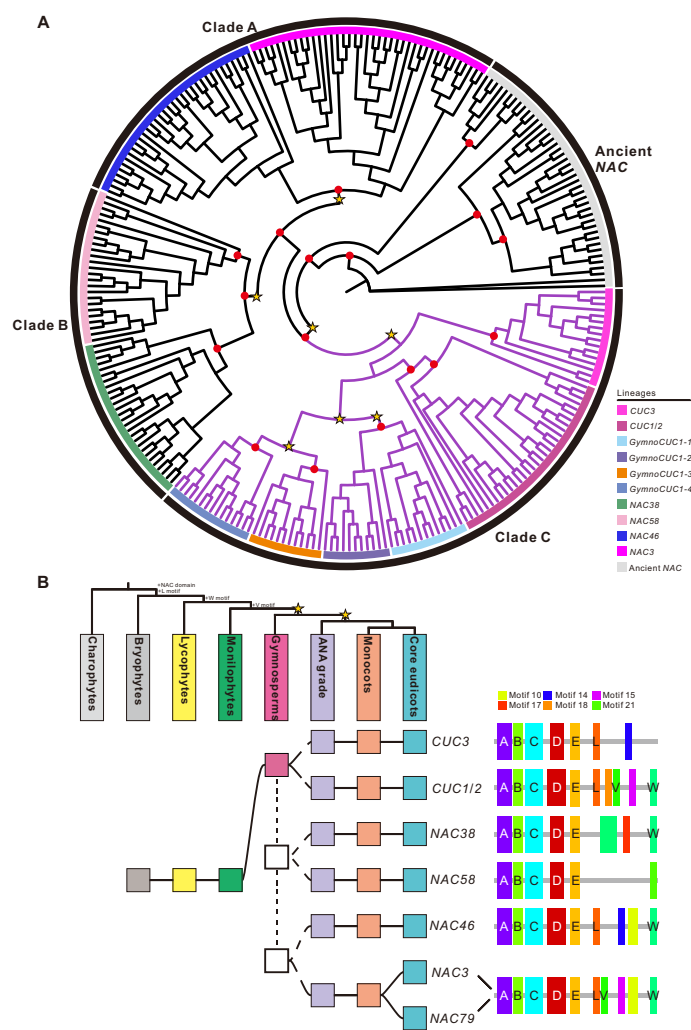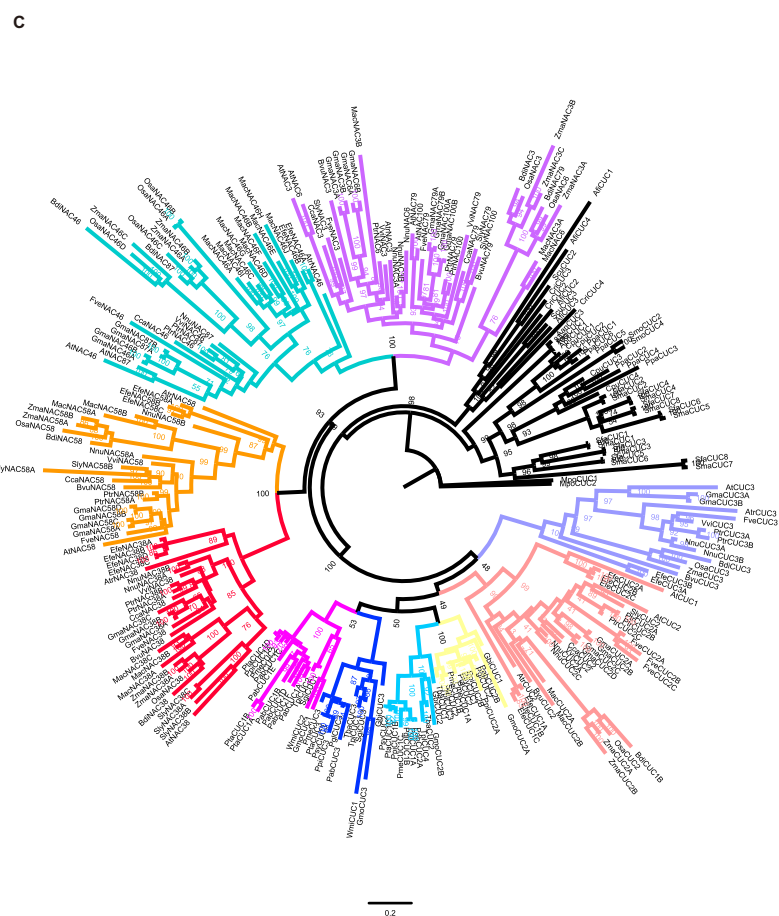

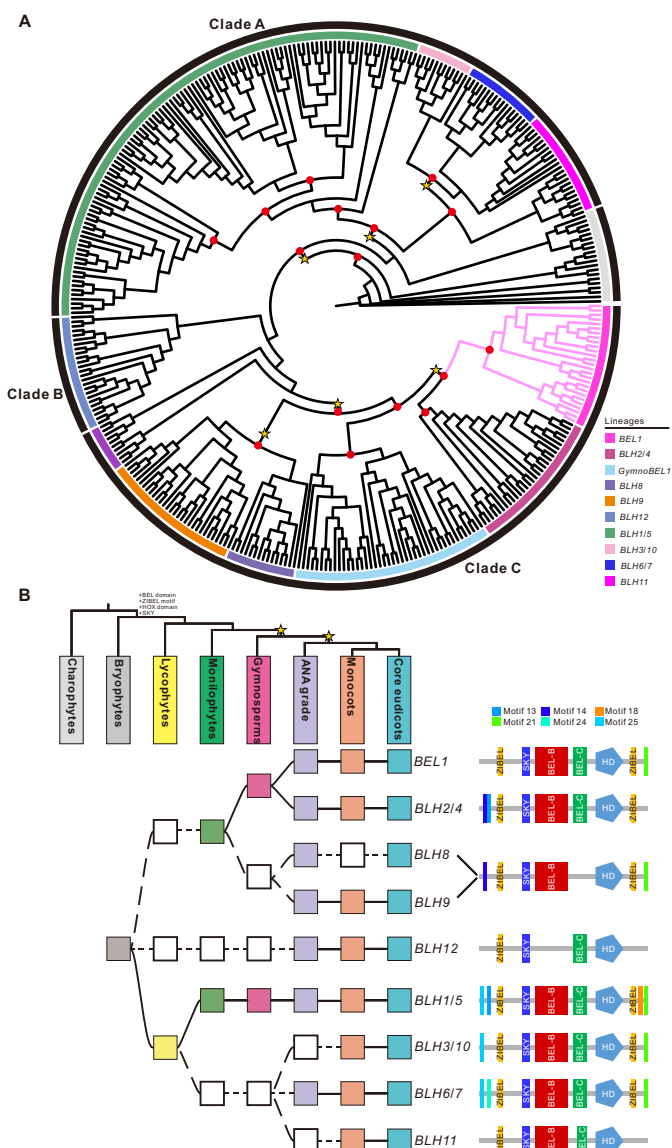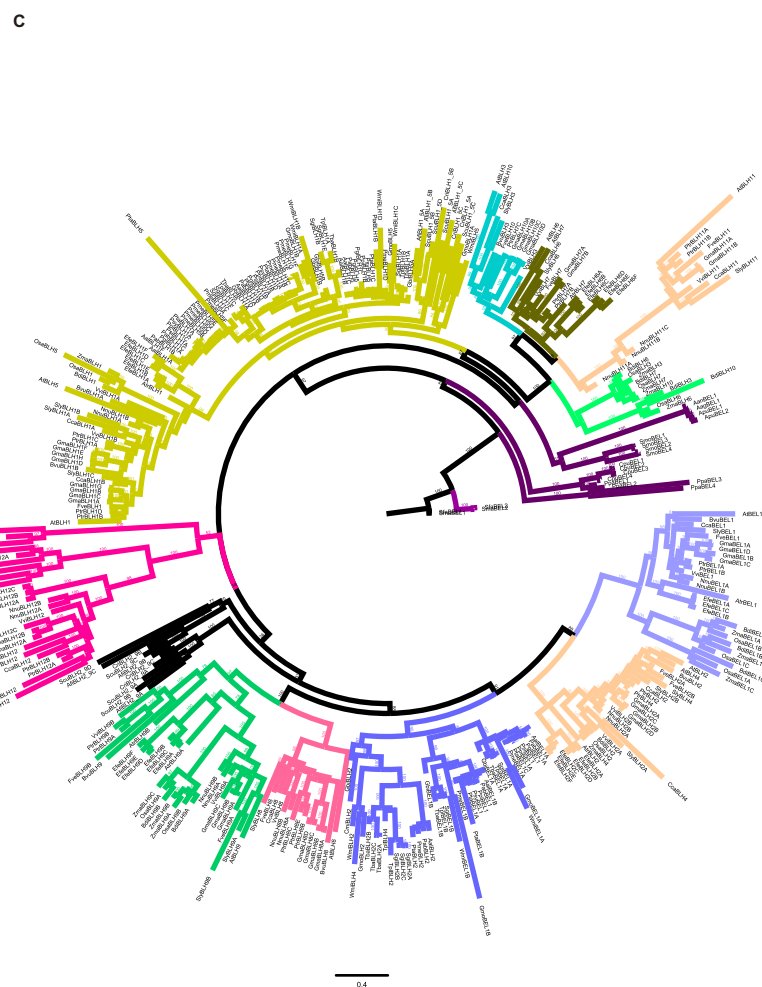

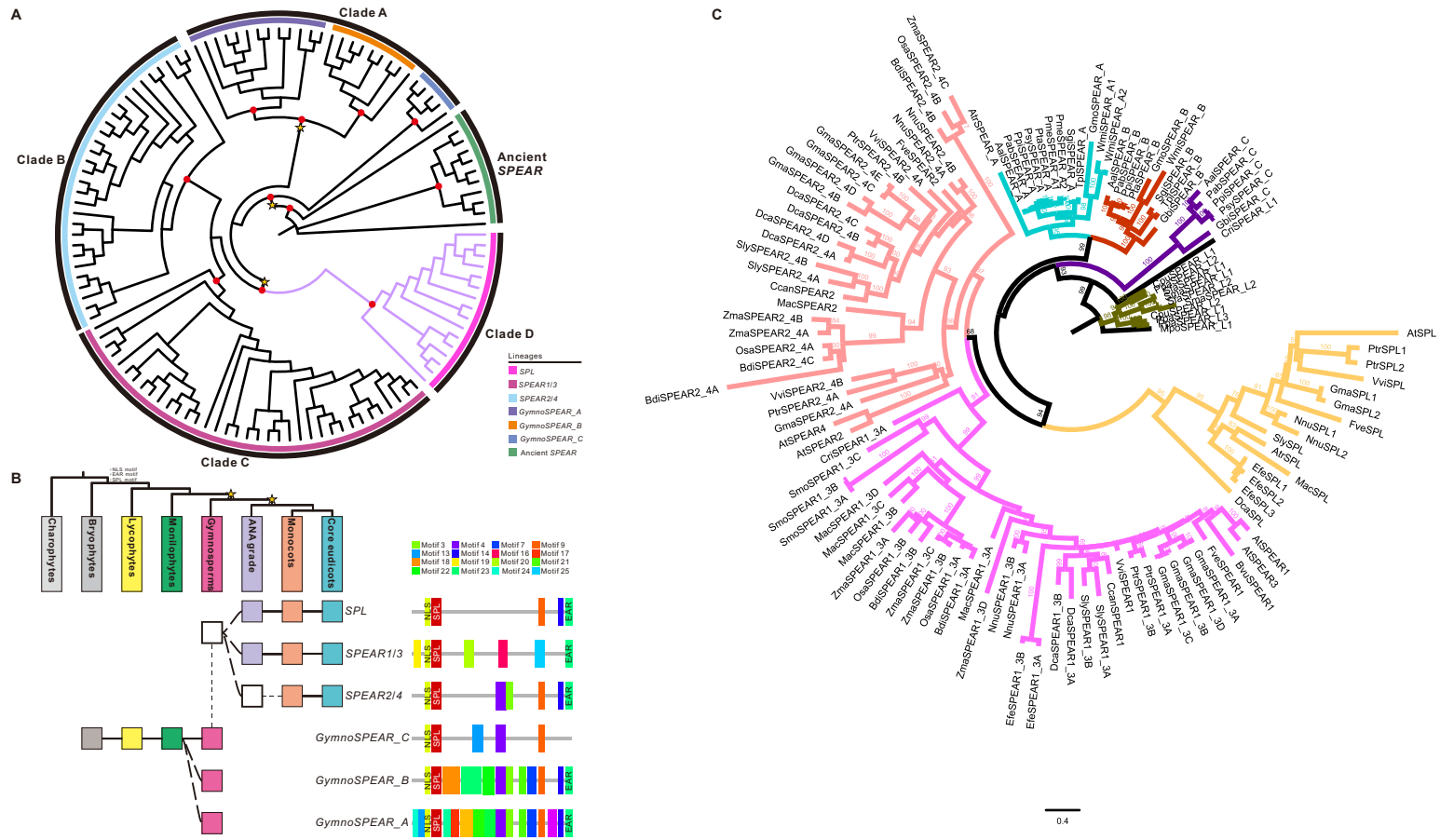

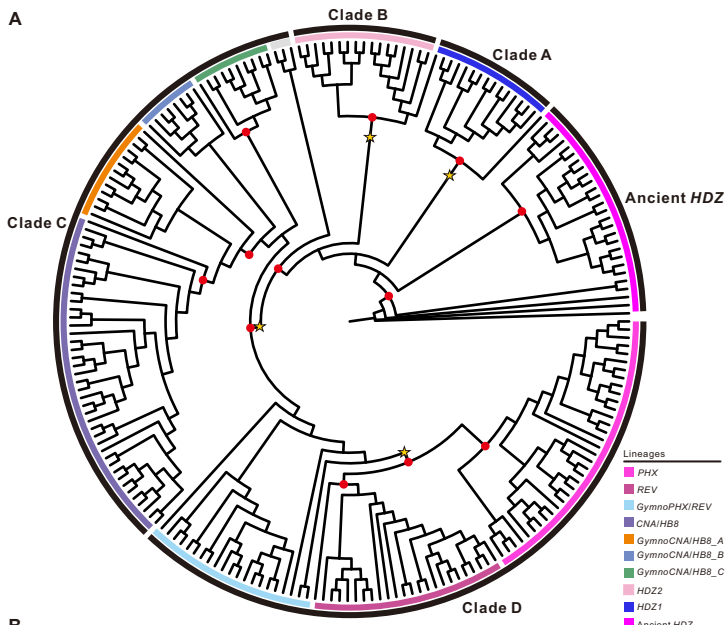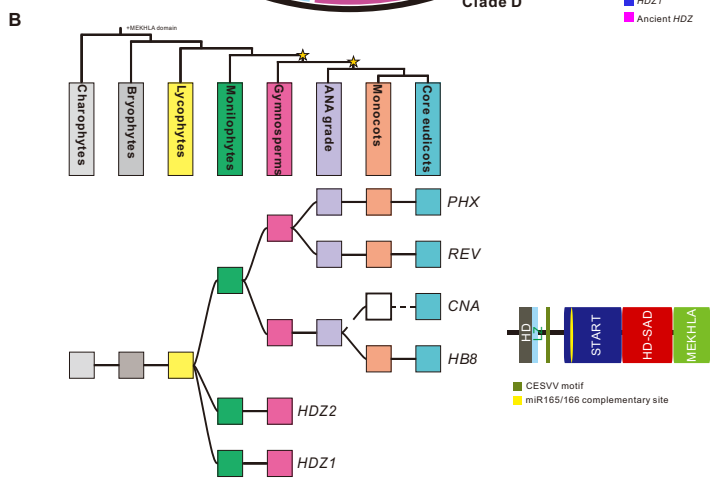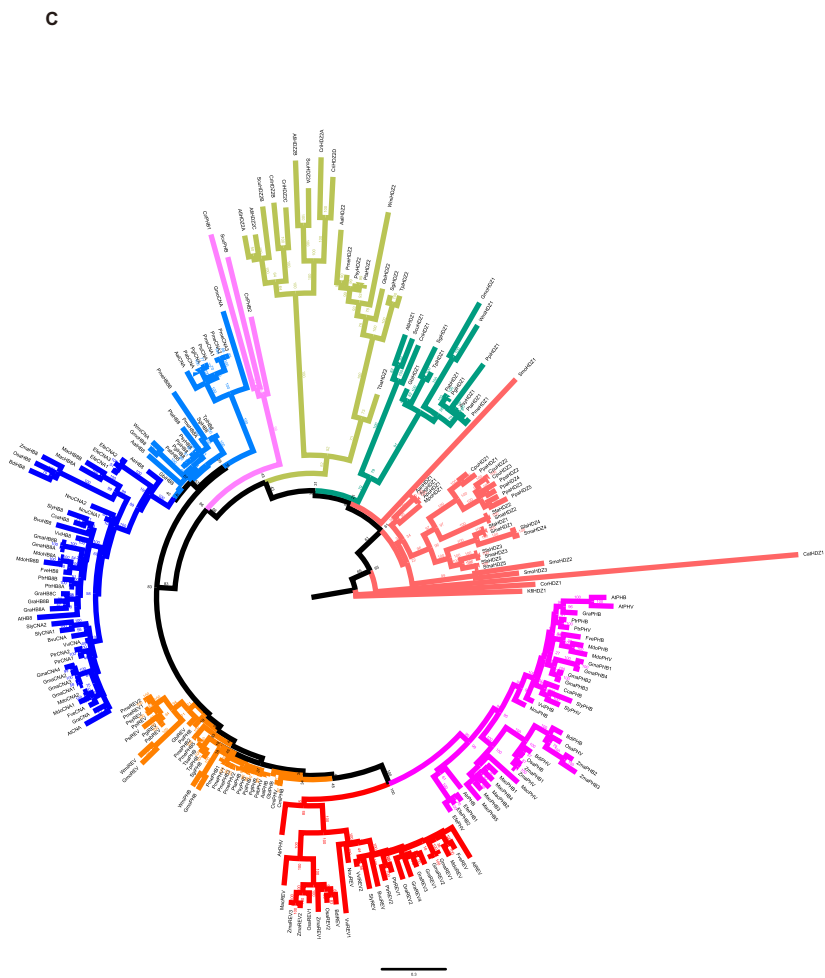

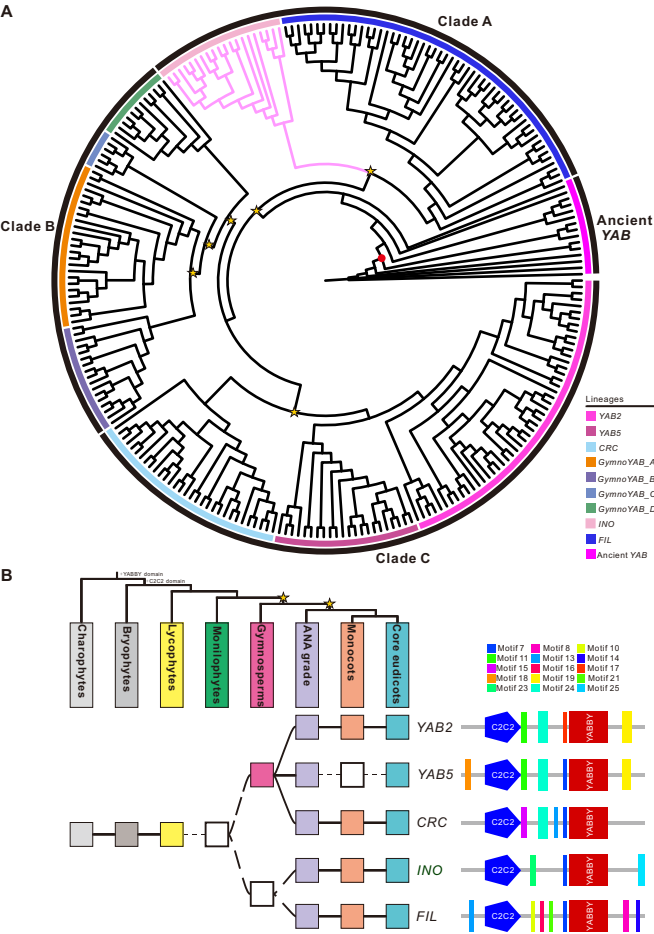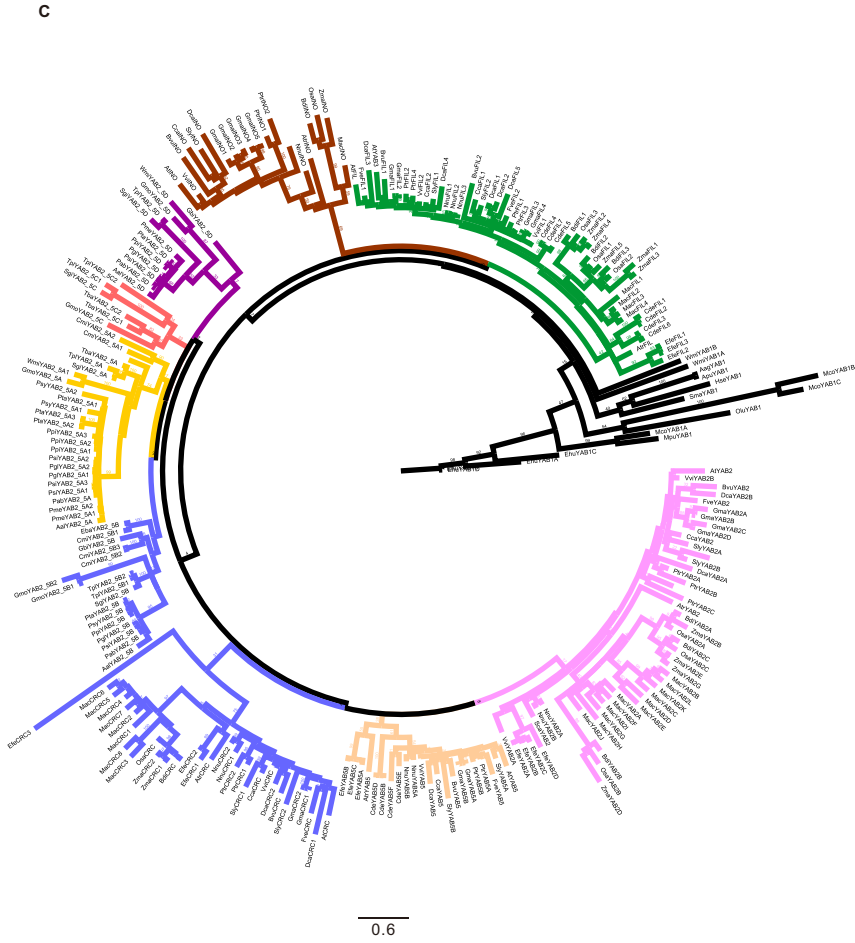

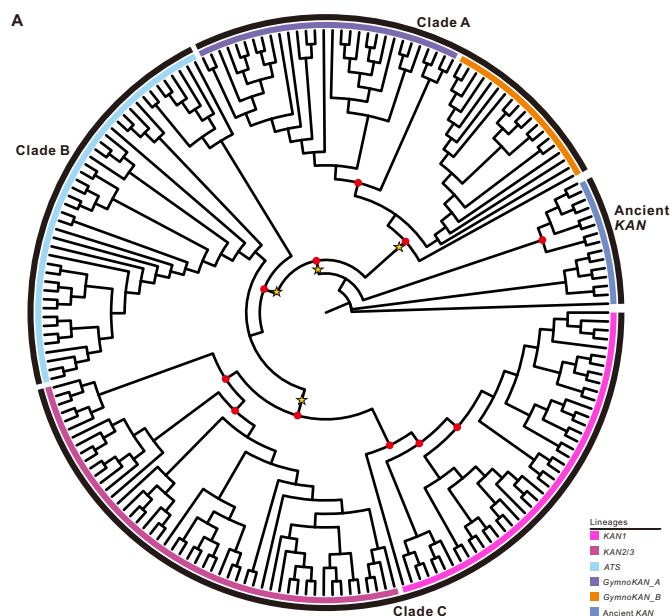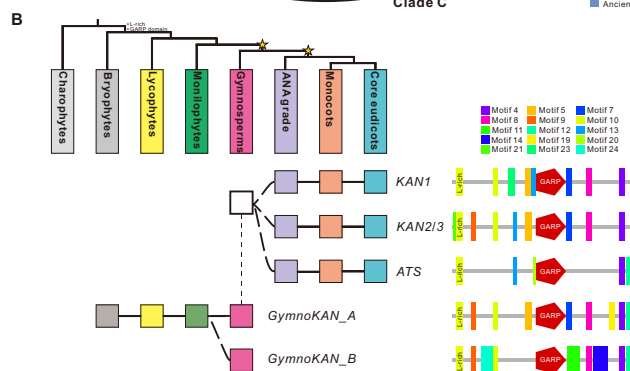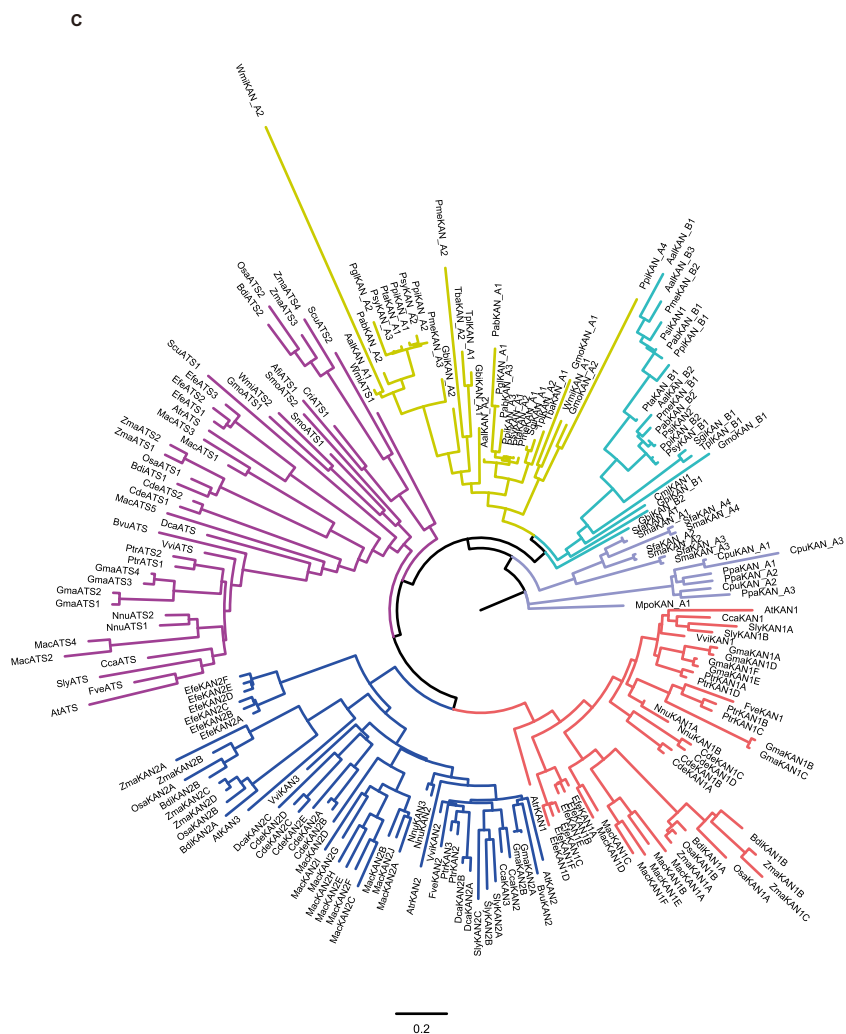

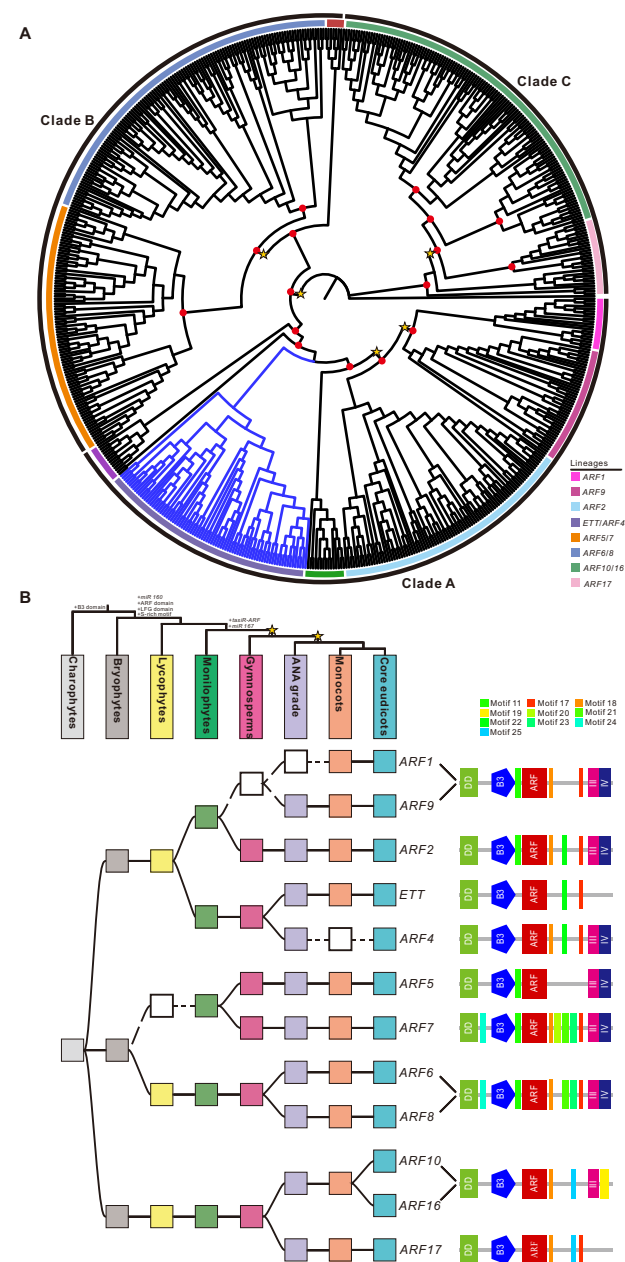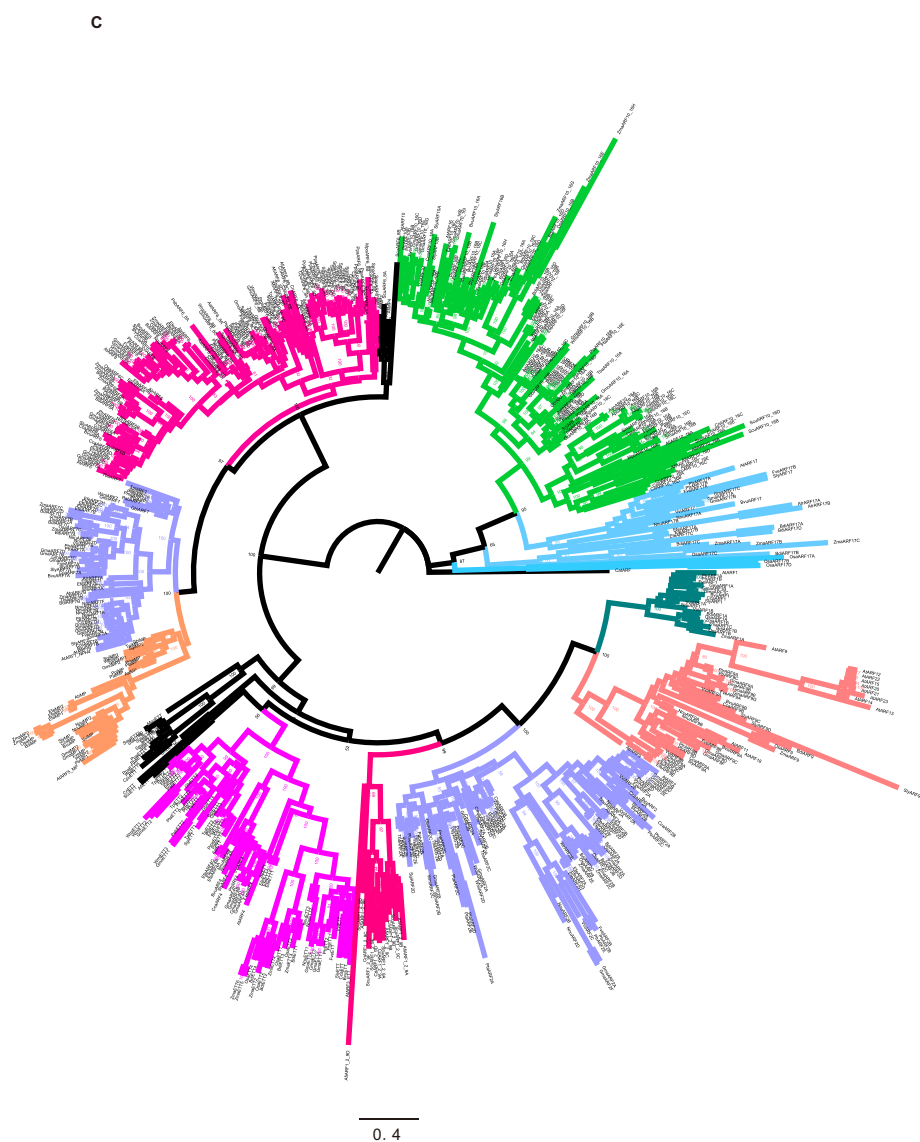

Supplement: Supplementary Figure 1 — Phylogeny and domain architecture of different gene families. Support values are shown for nodes. The scale bar indicates the number of changes per site. Different gene lineages are marked with different colors. (S1-1) Phylogeny and domain architecture of the ANT homologs. (A) Phylogenetic tree of 354 ANT homologs identified from diverse land plants. The red dot indicates the branch support value of BP > 85, while the yellow stars indicate the large-scale duplication events. Branches marked with pink indicate the ANT orthologs. The outer black circles represent the range of different clades, and the inner colored circles indicate sublineages within each clade. (B) Duplication history and domain architecture of the ANT homologs. Filled squares indicate the presence of the corresponding members, open squares indicate absence data. The color of squares is corresponding to the top organismal tree. The yellow stars in the tree indicate whole-genome duplication events. The diagram on the right demonstrates the domain/motif composition of different duplicates. The known conserved domains/motifs included: euANT1, euANT2, euANT3, euANT4, the AP2 domain (R1 and R2) and the linker regions (L). The unnamed domains/motifs are linage-specific and are marked with the colors corresponding to the sequence logos in Supplementary Figure S2-1 . (C) Phylogenetic tree of ANT homologs with the support values at each node. (S1-2) Phylogeny and domain architecture of the CUC homologs. (A) Phylogenetic tree of 266 CUC homologs identified from diverse land plants. The red dot indicates the branch support value of BP > 85, while the yellow star indicates the large-scale duplication events. Branches marked with purple indicate the CUC orthologs. The outer black circles represent the range of different clades and the inner colored circles indicate sublineages within each clade. (B) Duplication history and domain architecture of the CUC homologs. Filled squares indicate the presence of the corres [file DataSheet_1.pdf]

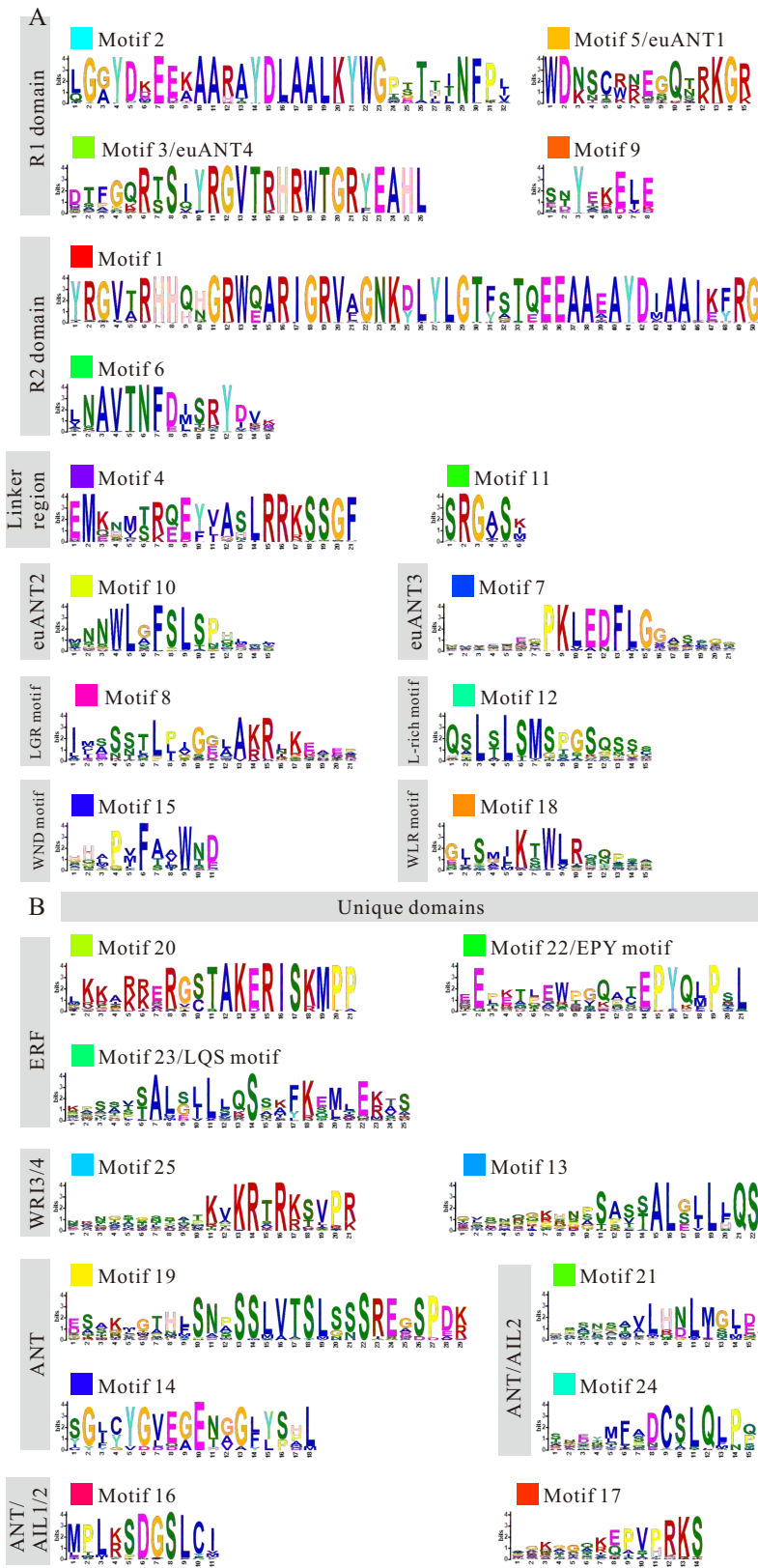

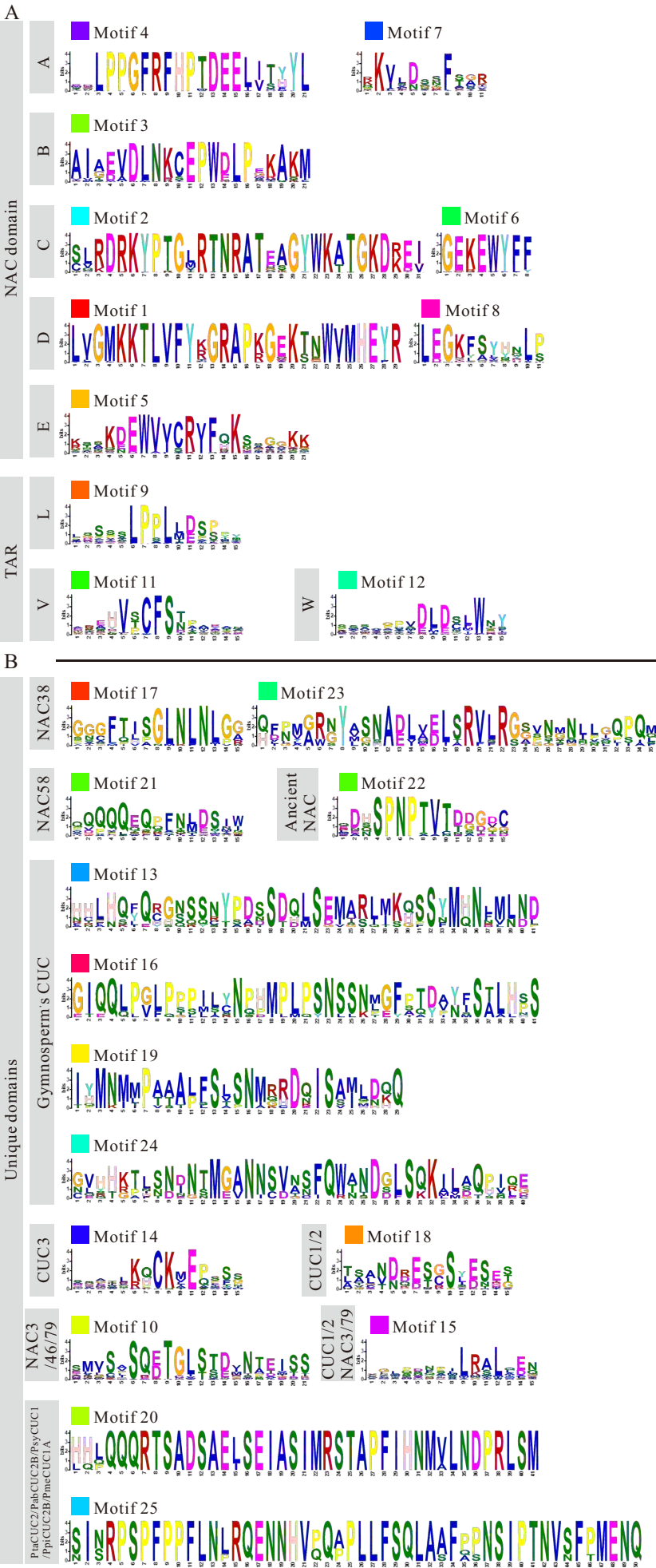

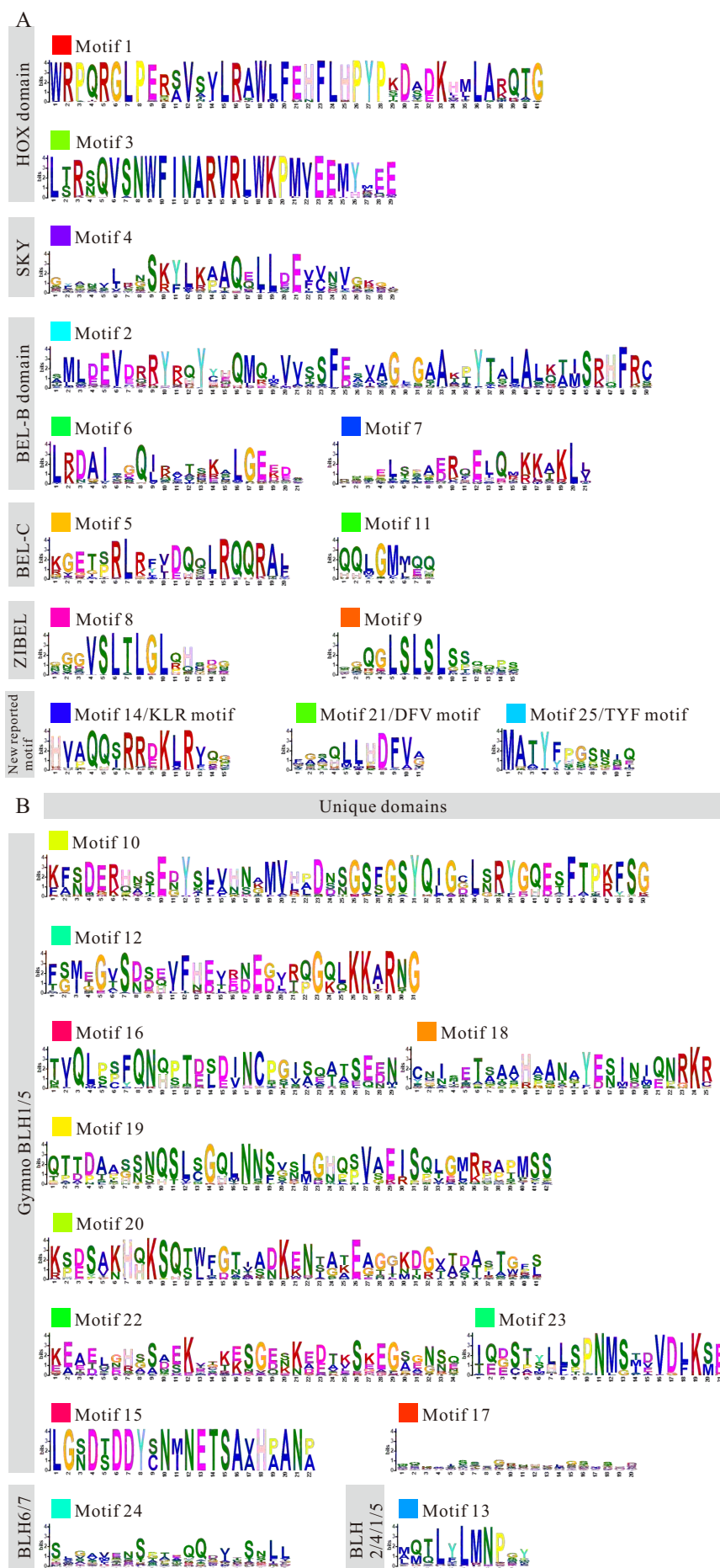

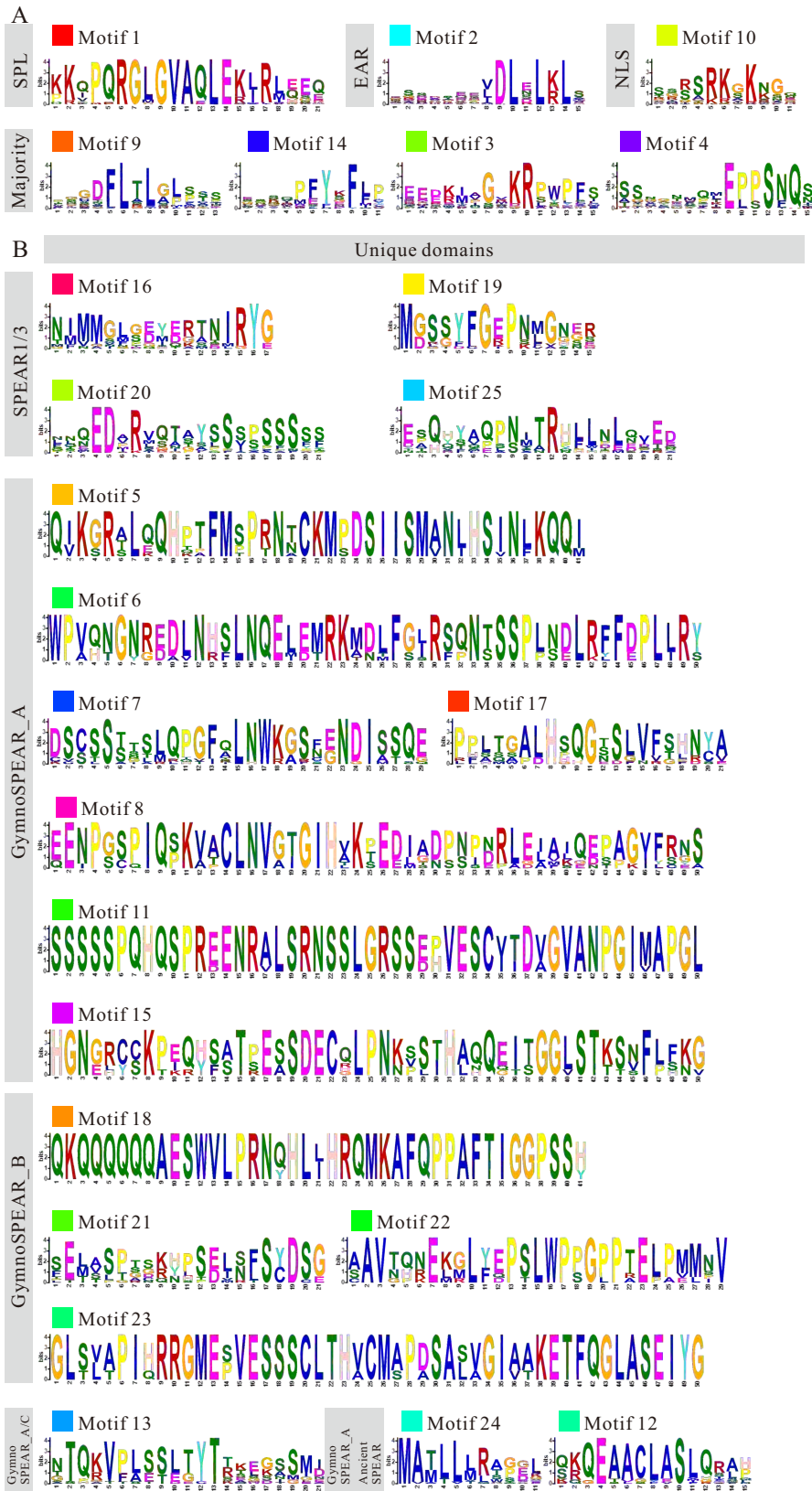

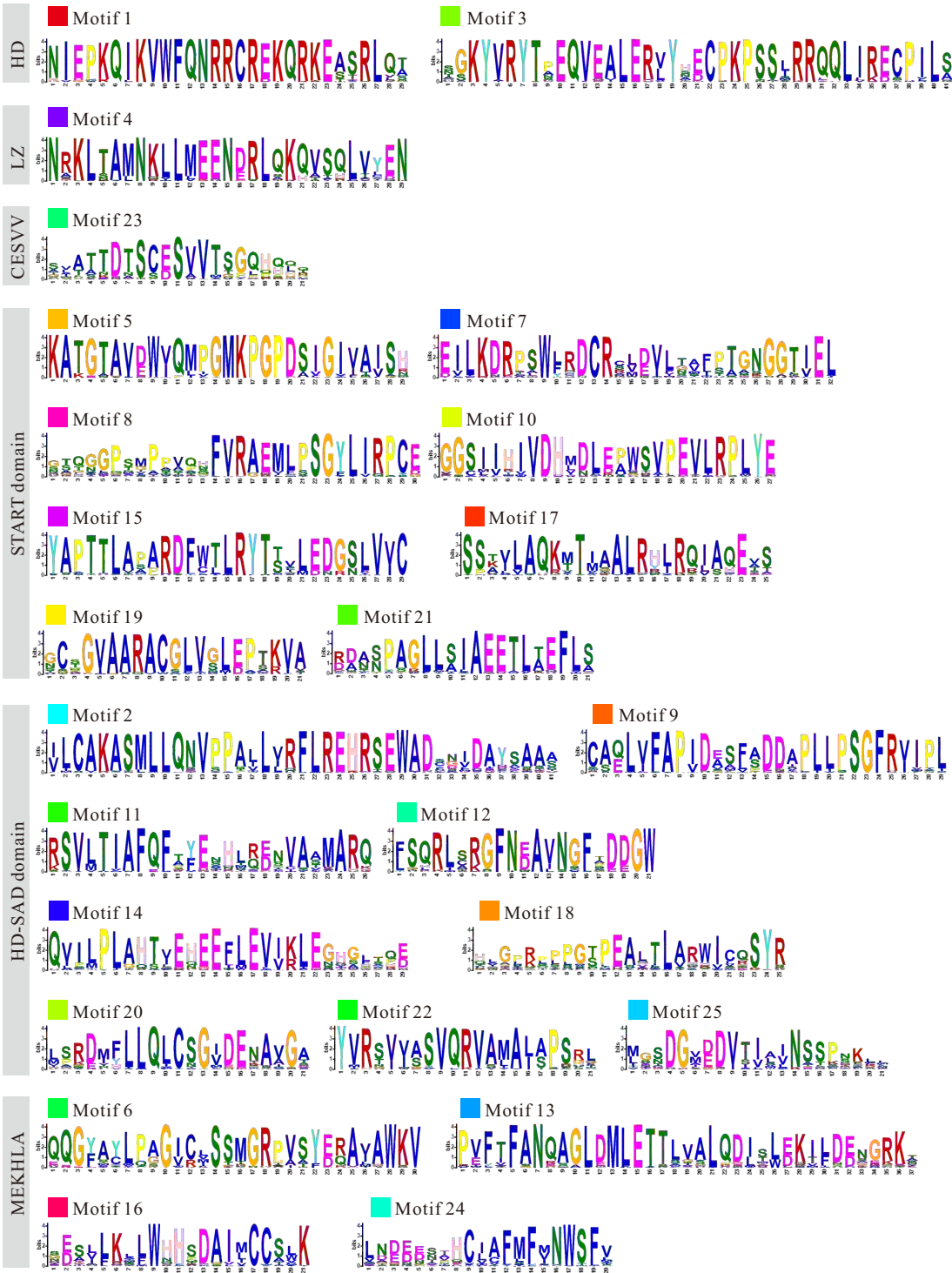

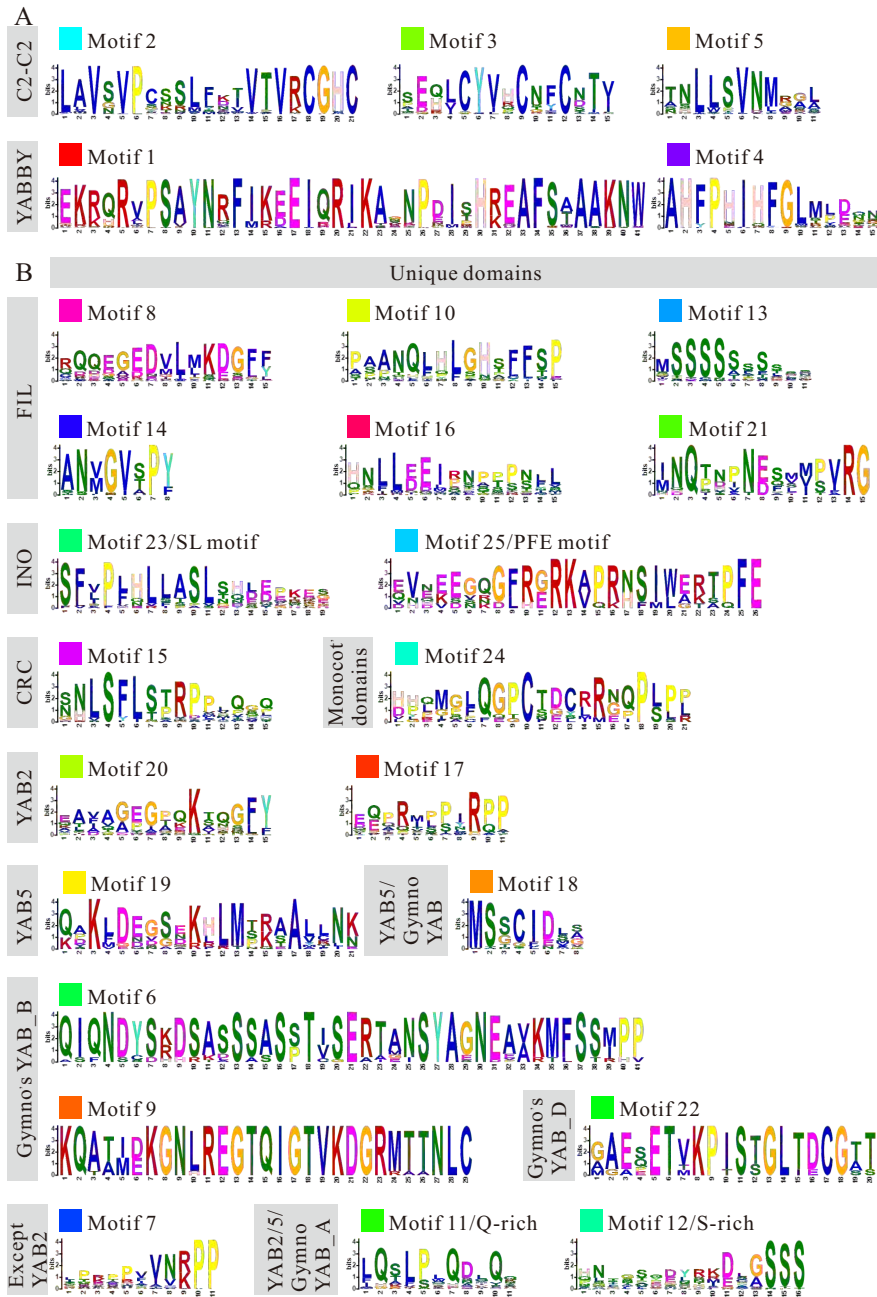

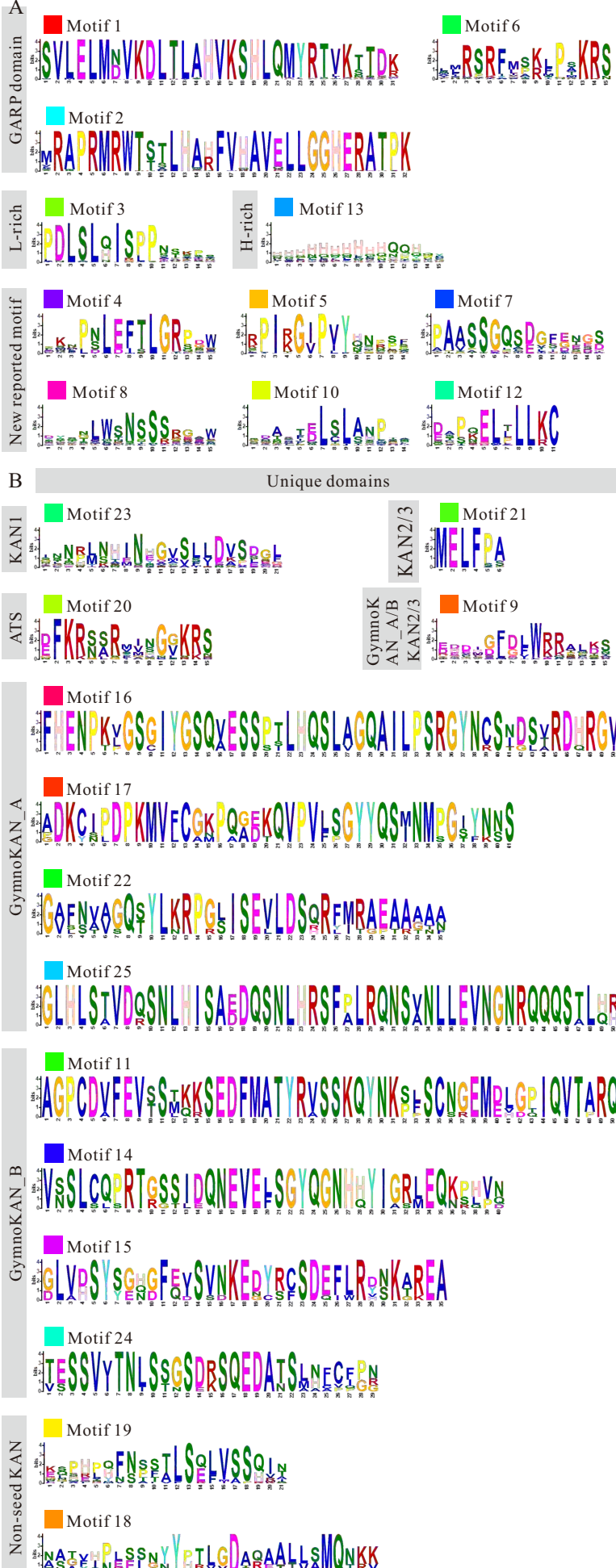

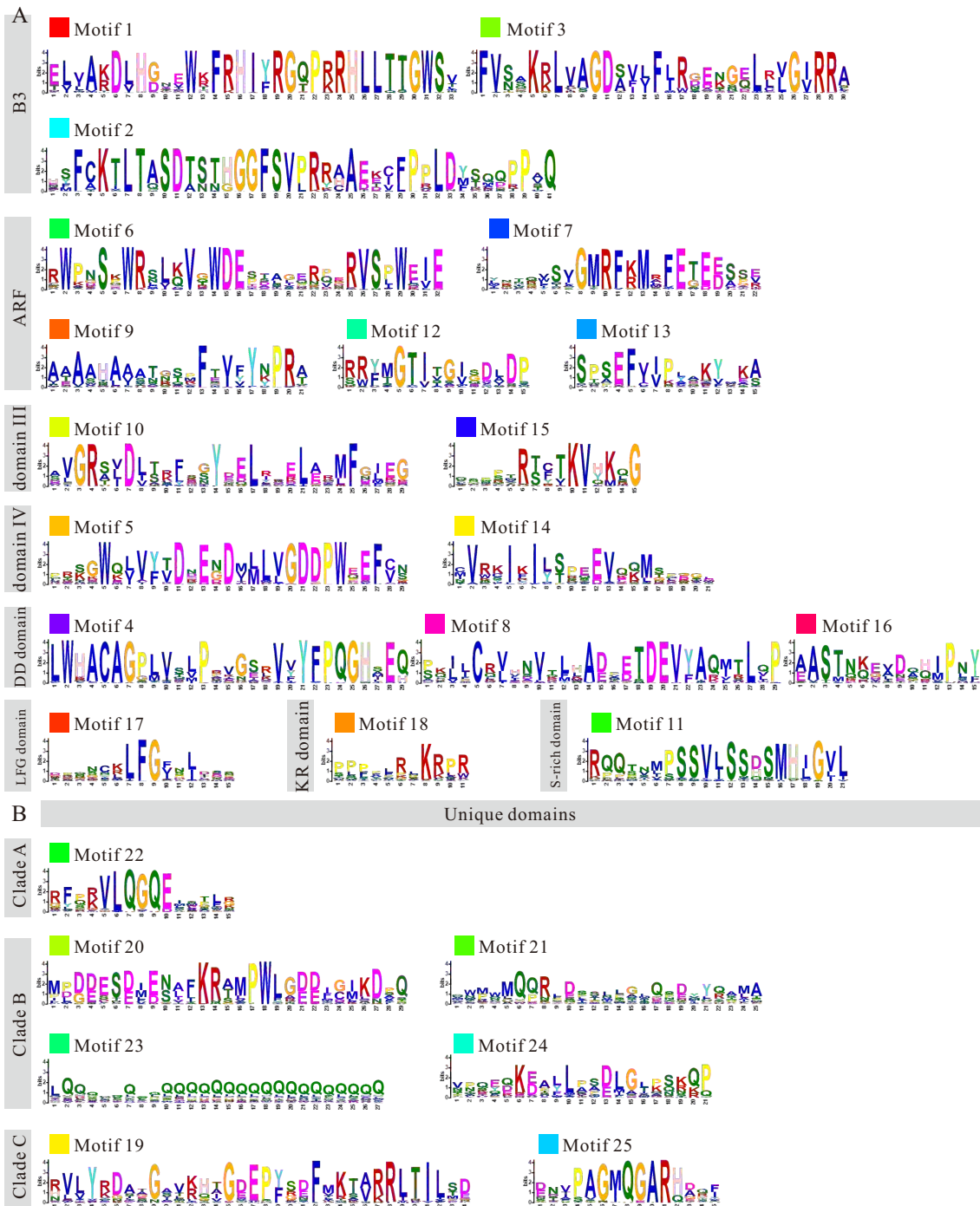

Supplement: Supplementary Figure 2 — Sequence logos of the conserved (A) and unique (B) domains/motifs of different gene families. The height of the letter indicates its relative frequency at the given position (x -axis) in the domain/motif. [file DataSheet_2.pdf]

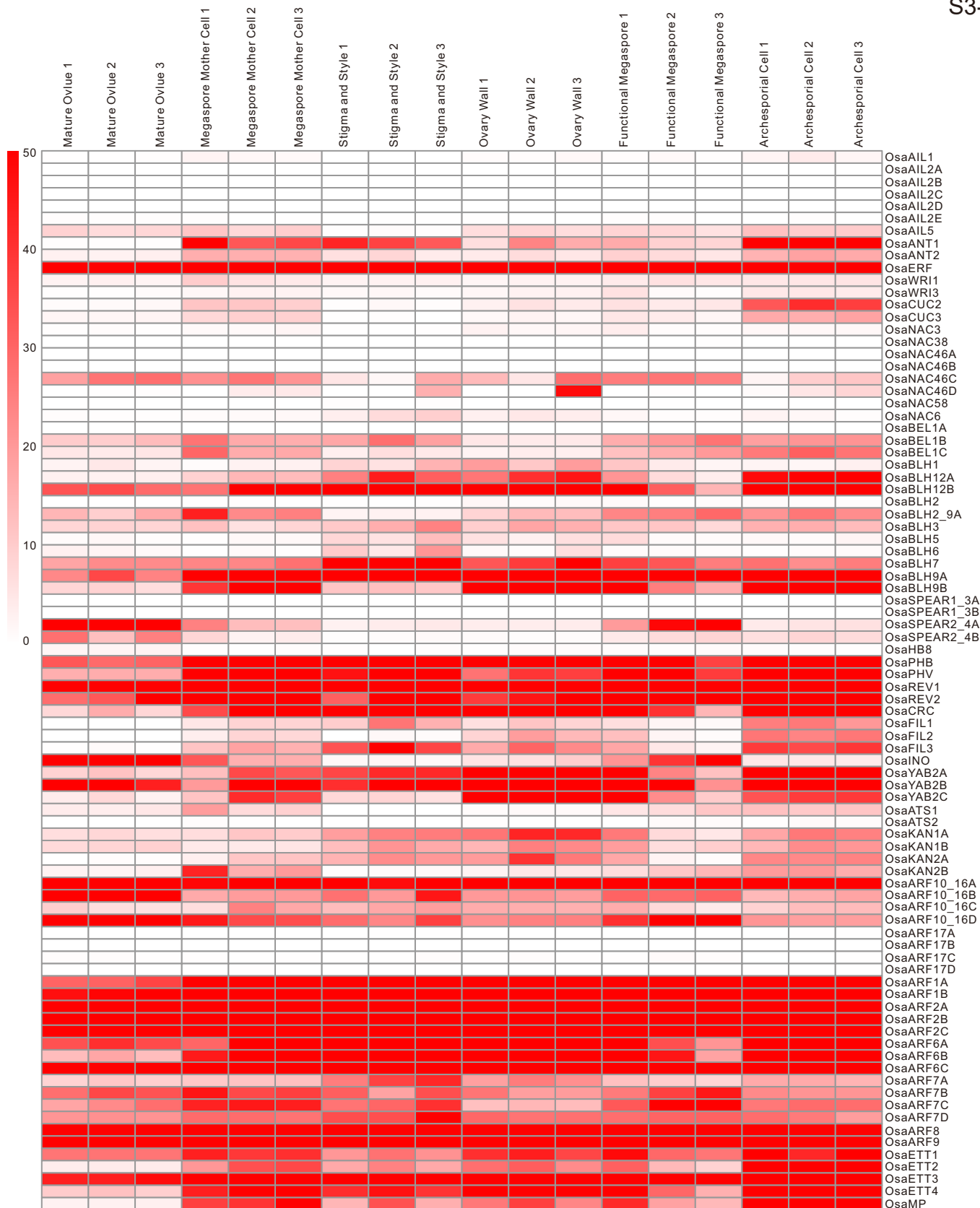

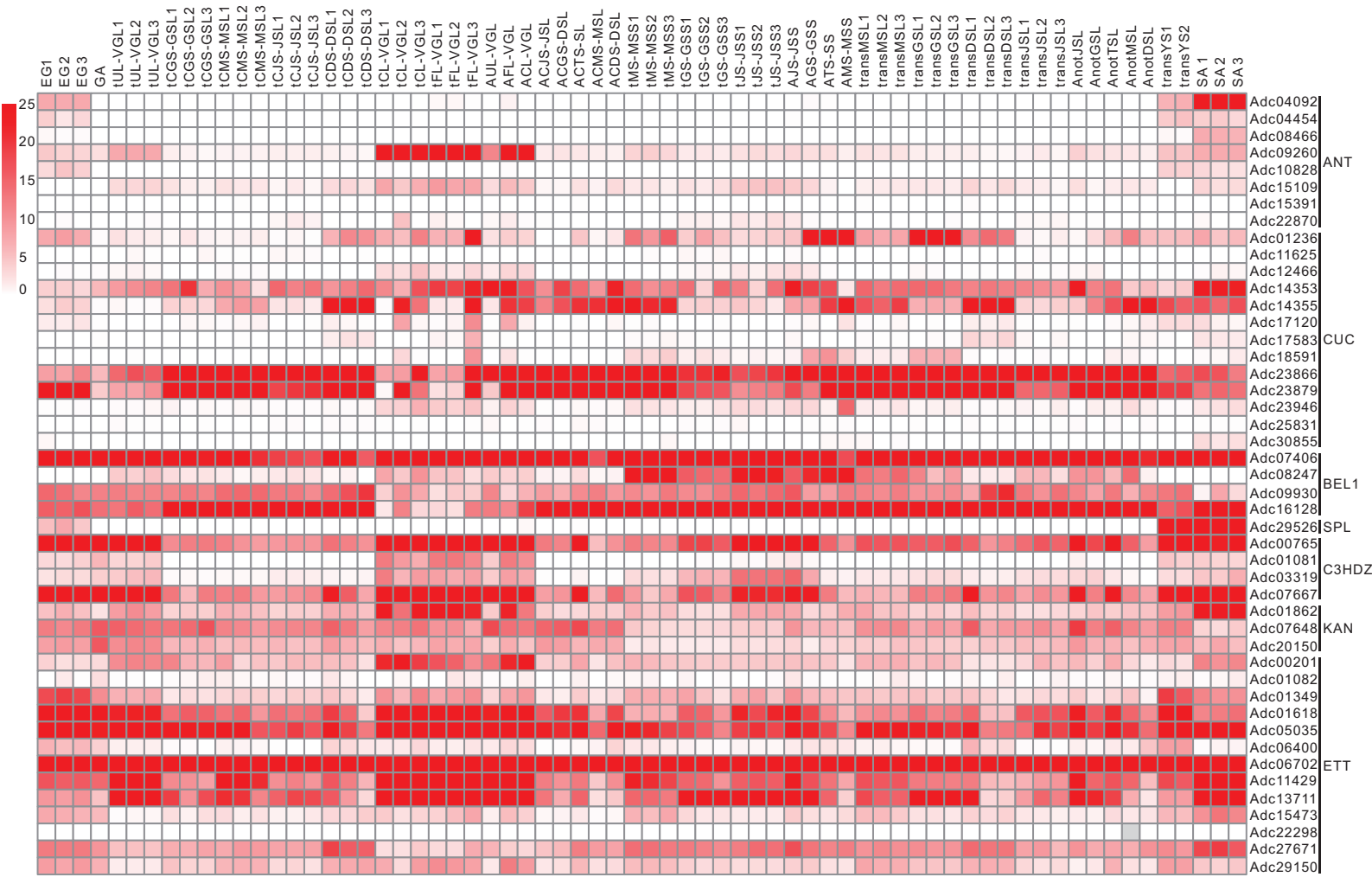

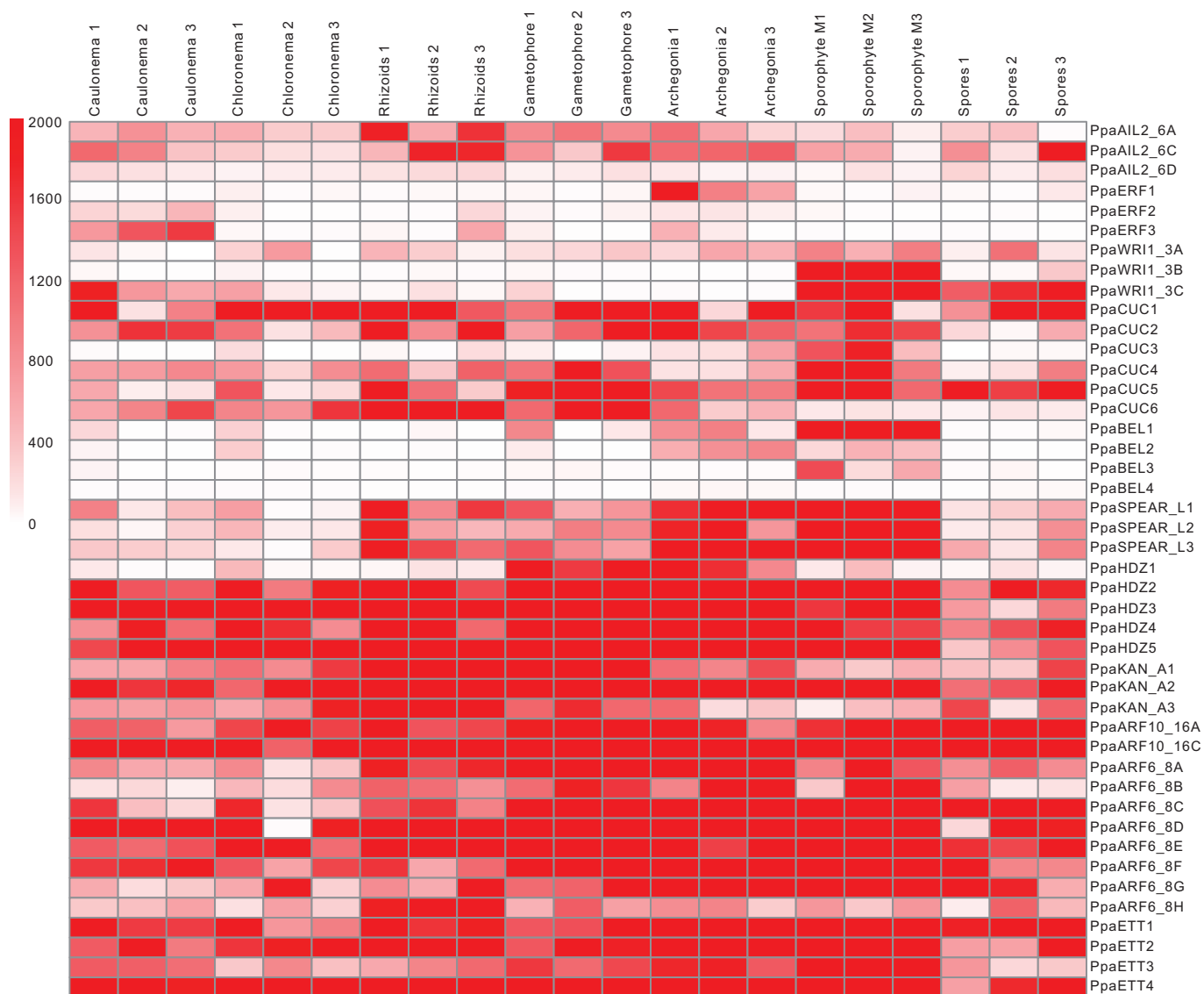

Supplement: Supplementary Figure 3 — Expression of key genes related to integument development in rice (S3-1), Adiantum capillus (S3-2) and Physcomitrella patens (S3-3). The 33 various developmental stages or tissues of A. capillus (S3-2) included the embryo gametophyte (EG), gametophyte (GA), the transUL vegetative growth leaf (tUL-VGL), the transLCGS green sporangium leaf (tCGS-GSL), the transLCMS mature sporangium leaf (tCMS-MSL), the transLCJS juvenile sporangium leaf (tCJS-JSL), the transLCDS dehiscent sporangium leaf (tCDS-DSL), the transCL vegetative growth leaf (tCL-VGL), the transFL vegetative growth leaf (tFL-VGL), the AnotUL vegetative growth leaf (AUL-VGL), the AnotFL vegetative growth leaf (AFL-VGL), the AnotCL vegetative growth leaf (ACJS-JSL), the AnotLCGS vegetative growth leaf (ACGS-DSL), the AnotLCTS sporangium leaf (ACTS-SL), the AnotLCMS mature sporangium leaf (ACMS-MSL), the AnotLCDS dehiscent sporangium leaf (ACDS-DSL), the transMS mature sporangium stage (tMS-MSS), the transGS green sporangium stage (tGS-GSS), the transJS juvenile sporangium stage (tJS-JSS), the AnotJS juvenile sporangium stage (AJS-JSS), the AnotGS green sporangium stage (ATS-SS), the AnotMS mature sporangium stage (AMS-MSS), the transMSL mature sporangium leaf (transMSL), the transGSL green sporangium leaf (transGSL), transDSL dehiscent sporangium leaf (transDSL), transJSL juvenile sporangium stage (transJSL), the AnotJSL juvenile sporangium stage (AnotJSL), the AnotGSL green sporangium leaf (AnotGSL), AnotTSL sporangium leaf (AnotTSL), the AnotMSL mature sporangium leaf (AnotMSL), the AnotDSL dehiscent sporangium leaf (AnotDSL), the transYS young sporophyte (transYS) and the stem apical (SA). [file DataSheet_3.pdf]
